# Advances in Hygroscopic Polymer Gels toward Efficient Atmospheric Moisture Capture and Management

**DOI:** 10.1002/advs.202518062

**Published:** 2025-11-16

**Authors:** Ruofan Zhou, Yini Hu, Peng Xiao, Jincui Gu, Tao Chen

**Affiliations:** ^1^ State Key Laboratory of Advanced Marine Materials Zhejiang Key Laboratory of Extreme‐environmental Material Surfaces and Interfaces Ningbo Institute of Material Technology and Engineering Chinese Academy of Science Ningbo 315201 P. R. China; ^2^ School of Chemical Sciences University of Chinese Academy of Sciences 19A Yuquan Road Beijing 100049 P. R. China; ^3^ College of Material Chemistry and Chemical Engineering Key Laboratory of Organosilicon Chemistry and Material Technology Ministry of Education Hangzhou Normal University Hangzhou 311121 P. R. China

**Keywords:** atmospheric moisture capture, hygroscopic mechanisms, hygroscopic polymer gel

## Abstract

Atmospheric humidity, as a widely distributed environmental resource, holds great potential to create significant value for human society via efficient regulation and utilization. Hygroscopic polymer gels (HPGs) have emerged as a highly promising material platform for atmospheric moisture capture, owing to their highly tunable structures, unique swelling behaviors, and versatile functionality. In this review, recent advances in HPGs, including the hygroscopic mechanisms and state‐of‐the‐art construction strategies, are systematically summarized. Furthermore, cutting‐edge applications of HPGs, including atmospheric water harvesting, electricity production, thermal management, fuel production, advanced greenhouses, dehumidification, and HPGs‐based electrolytes, are thoroughly discussed. The review places particular emphasis on the urgent need to intensify the design and development of HPGs specifically tailored for sustainable applications in challenging low‐humidity arid regions. Finally, the current challenges are summarized, and future research directions for next‐generation HPGs in atmospheric moisture management are outlined, aiming to fully harness the potential of atmospheric humidity as an abundant and renewable resource.

## Graphical abstract

This review systematically elaborates on the research progress of hygroscopic polymer gels (HPGs) in the field of atmospheric moisture management, summarizes their hygroscopic mechanisms, construction strategies, and typical multi‐field applications, highlights the urgency of developing moisture‐adaptive HPGs for low‐humidity arid regions, and explicitly outlines the current challenges and future research directions.

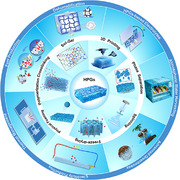

## 1 Introduction

Under the dual pressures of globally uneven water distribution and rising energy demands, atmospheric water has emerged as a pivotal resource. Widely distributed and continuously replenished by the global water cycle, it holds ≈12900 km^3^ of water in total—roughly six times the volume of all Earth's rivers combined. Efficient harvesting and utilization of atmospheric water thus represent a strategic approach to alleviating freshwater scarcity while advancing sustainable energy transitions.^[^ ^1^, ^2^ ^]^ An ideal hygroscopic material should possess high water adsorption capacity, rapid adsorption kinetics, low energy demand for desorption, and excellent cyclic stability.^[^ ^3^ ^]^ Diverse classes of hygroscopic materials, such as metal–organic frameworks (MOFs)^[^ ^4^, ^5^, ^6^ ^]^ and covalent organic frameworks (COFs),^[^ ^7^, ^8^, ^9^ ^]^ feature adjustable chemical structures that allow precise structural design. This endows them with diverse moisture adsorption characteristics to adapt to varied environmental conditions. However, their practical applications are limited by structural brittleness and insufficient heat and mass transfer performance.^[^ ^10^ ^]^ Conversely, hygroscopic organic liquids (e.g., glycerol^[^ ^11^, ^12^ ^]^ and ionic liquids^[^ ^13^, ^14^, ^15^ ^]^) and salts^[^ ^16^, ^17^, ^18^, ^19^ ^]^ exhibit excellent sorption capacities across a wide range of relative humidity (RH) owing to their multistep sorption mechanism. Nevertheless, their moisture adsorption capacity is highly dependent on RH. Furthermore, some salt‐based materials are prone to deliquescence at high RH, structural disintegration, particle agglomeration, and ultimately porous network collapse–severely compromising both sorption efficiency and cycling durability.^[^ ^1^ ^]^

To overcome the inherent limitations of single‐component hygroscopic materials, a promising strategy is to encapsulate organic liquids and salts in hygroscopic polymer gels (HPGs). This design leverages the tunable structural parameters, physicochemical properties, hierarchical pore topology, swelling behavior, and efficient interfacial integration capabilities of the gel matrix. Specifically, the high‐surface‐area networks provide nanoscale anchoring sites for the dispersion of salt particles, effectively mitigating agglomeration and deactivation of the hygroscopic components. Simultaneously, interconnected meso‐ and macropores establish continuous pathways for vapor diffusion, significantly reducing mass transfer resistance. Furthermore, capillary confinement of hygroscopic liquids improves solution retention and enhances cyclic stability.^[^ ^20^ ^]^ Although recent progress in HPGs research has encompassed functional motif engineering, studies focusing on low‐humidity applications remain in their nascent stages.^[^ ^21^ ^]^ Substantial challenges also persist in optimizing material performance and expanding application boundaries.^[^ ^22^, ^23^, ^24^, ^25^ ^]^ A comprehensive review is therefore essential to elucidate the structure–property relationships in existing HPGs.

**Figure 1:**
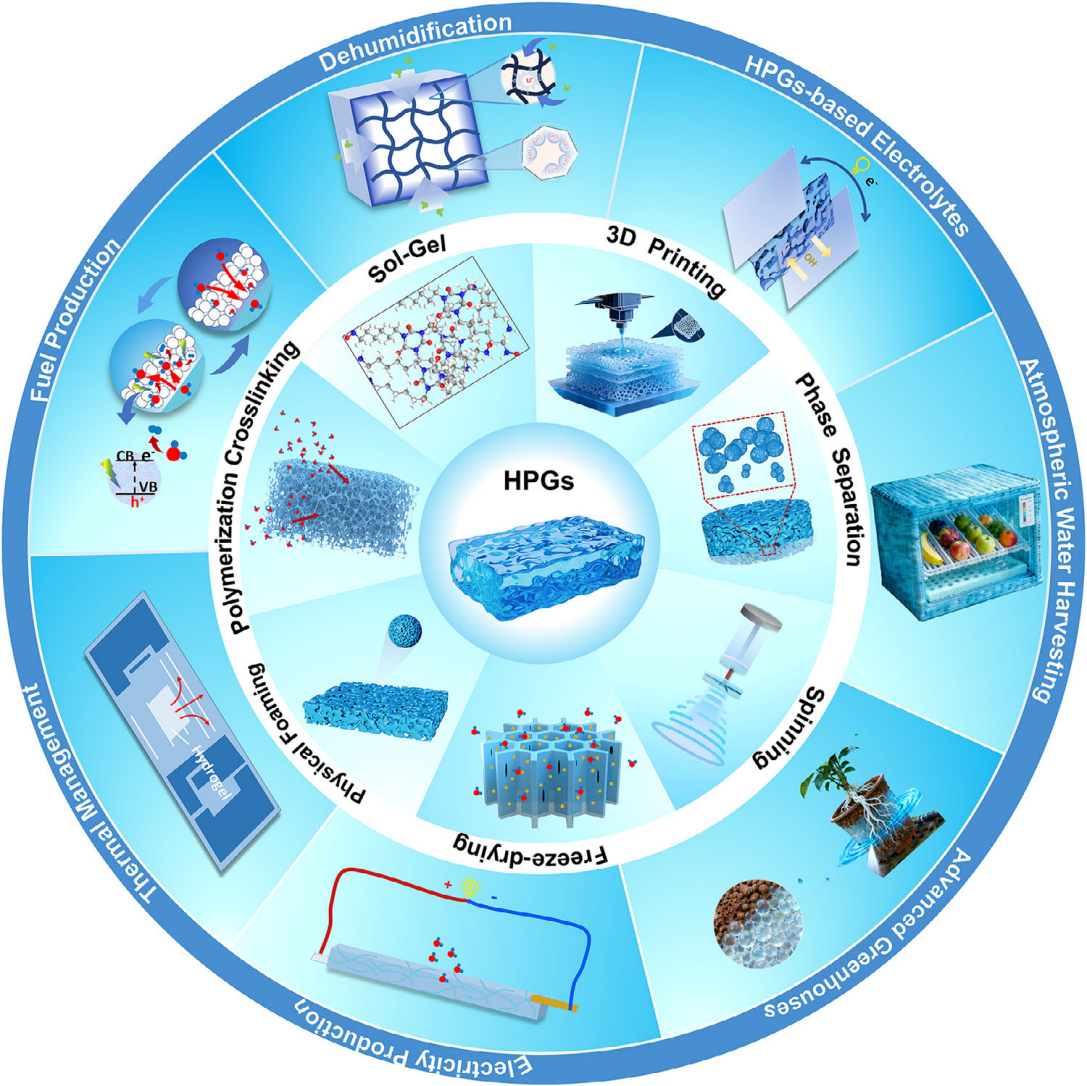
Overview of fabrication strategies and cutting‐edge applications of HPGs. This review focuses on state‐of‐the‐art fabrication strategies for HPGs, including sol‐gel, polymerization crosslinking, freeze‐drying, 3D printing, physical foaming, phase separation, and spinning.

In this context, we present a comprehensive review of emerging HPGs for advanced atmospheric moisture management. The analysis begins with an in‐depth examination of hygroscopic mechanisms in HPGs, supported by thermodynamic and kinetic frameworks. Subsequently, we systematically categorize structural engineering methodologies—including sol‐gel,^[^ ^26^ ^]^ polymerization crosslinking,^[^ ^27^ ^]^ physical foaming,^[^ ^28^ ^]^ freeze‐drying,^[^ ^29^ ^]^ spinning,^[^ ^30^ ^]^ phase separation,^[^ ^31^ ^]^ and 3D printing^[^ ^32^ ^]^—while critically evaluating their design principles. Through critical analysis of seminal studies, the review highlights cutting‐edge designs that precisely regulate water‐material interactions to enable atmospheric moisture management for enhanced capture efficiency. Moreover, the review showcases a spectrum of state‐of‐the‐art applications of HPGs in freshwater generation—such as atmospheric water harvesting (AWH),^[^ ^33^ ^]^ electricity generation,^[^ ^34^ ^]^ thermal management,^[^ ^35^ ^]^ fuel production,^[^ ^36^ ^]^ advanced greenhouses,^[^ ^37^ ^]^ dehumidification,^[^ ^38^ ^]^ and HPGs‐based electrolytes.^[^ ^39^ ^]^ These applications, while promising, face unique challenges in extreme environments, with particular emphasis placed on the pressing need to develop sustainable HPGs tailored for diverse applications in arid, low‐humidity regions. By delineating current challenges and charting actionable future directions, this work aims to guide the development of next‐generation HPGs (**Figure** **1**).

## 2 Atmospheric Moisture Absorption/Desorption Mechanism of HPGs

### 2.1 Thermodynamics Mechanism of HPGs

**Figure 2:**
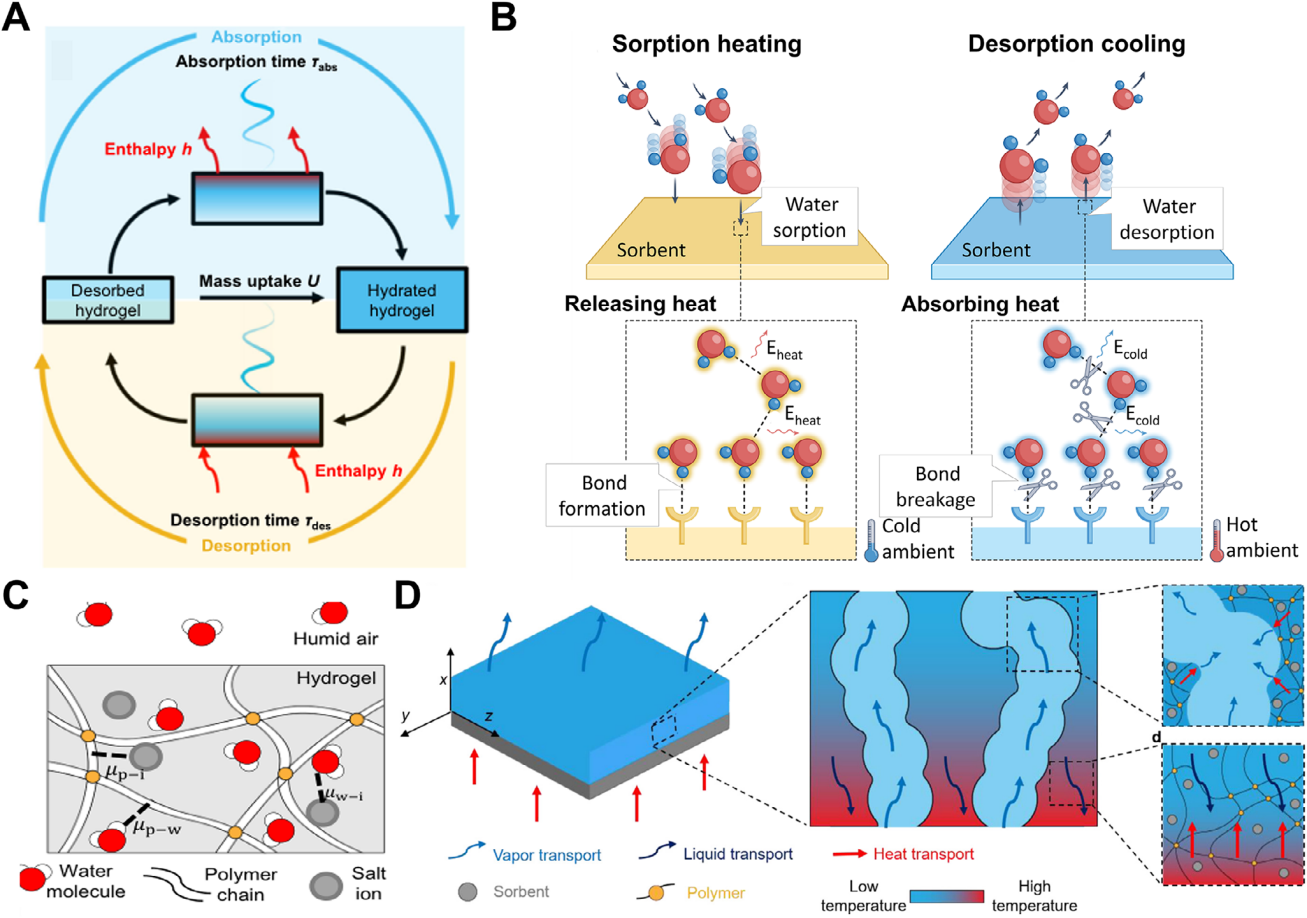
Thermodynamics Mechanism of HPGs. A) The absorption/desorption mechanism of HPGs, and the bridging the gap between hydrogel‐salt composite composition and absorption properties to optimize application performance. Reproduced with permission.^[^ ^40^ ^]^ Copyright 2024, Springer Nature. B) Sorbents capture water from the ambient air with bond formation, and Water escapes from sorbents to the ambient air with bond breakage. Reproduced with permission.^[^ ^41^ ^]^ Copyright 2024, Springer Nature. C) Polymer‐water, water‐ion, and polymerion interactions dictate the chemical potential of water in the hydrogel, which, in equilibrium, equals the chemical potential of the water in the air. Reproduced with permission.^[^ ^40^ ^]^ Copyright 2024, Springer Nature. D) Thermally‐driven desorption of hygroscopic hydrogels. Reproduced with permission.^[^ ^42^ ^]^ Copyright 2022, Elsevier.

The moisture absorption and desorption in HPGs represent a coupled thermodynamic process involving mass transfer, energy conversion, and interfacial phenomena. This process includes not only the diffusion and transport of water molecules within the polymer network, but also their interaction with polar functional groups, accompanied by energy exchange during vapor adsorption and desorption (**Figure** **2**A).^[^ ^40^ ^]^ During moisture absorption, water molecules form chemical bonds or van der Waals interactions with the adsorbent, releasing significant heat—an exothermic effect whose intensity depends on the adsorbent's water‐sorption enthalpy and moisture absorption capacity. Similarly, the water desorption process shows a considerable endothermic effect caused by the breaking of bonds or van der Waals forces(Figure 2B).^[^ ^41^ ^]^ The generated energy comes from the phase conversion of water between the high‐enthalpy and high‐entropy gaseous state and the low‐enthalpy and low‐entropy adsorbed state.

#### 2.1.1 Thermodynamic Mechanism of the Moisture Adsorption/Desorption Process

Recent advances in non‐equilibrium thermodynamics have enabled a deeper understanding of the correlation between Gibbs free energy and chemical potential during moisture sorption. Specifically, this progress has clarified how core thermodynamic concepts (e.g., chemical potential and Gibbs free energy) regulate the dynamic process of moisture sorption.^[^ ^43^ ^]^

To understand the interactions between polymers, water, and ions, a thermodynamic framework for hydrogel‐salt composites was developed based on the Flory‐Rehner theory for hydrogel swelling. The change in Gibbs free energy (*ΔG*) for the hydrogel‐salt composite can be expressed as Equation (1):

(1) $$\Delta G=-T\Delta S_{mix}+\Delta H_{mix}+\Delta G_{elas}$$

where *T* is the absolute temperature, *ΔS_mix_* is the mixing entropy between the polymer, water, and ions, *ΔH_mix_* is the mixing enthalpy between the polymer, water, and ions, and *ΔG_elas_* is the Gibbs free energy resulting from the elastic expansion of the hydrogel.

Chemical potential (*µ*), a central concept in thermodynamics, represents the driving force for substance transfer. It reflects differences in the energy state of a substance across regions or phases and dictates the direction and tendency of mass migration. For hygroscopic behavior, researchers formulated an expression for the chemical potential of water (Figure 2C; Equation (2)):

(2) $$\mu_{w,hydrogel}=\mu_{w}^{0}+\frac{\partial \Delta G}{\partial c_{w}}=\mu_{w}^{0}+\mu_{p-w}+\mu_{w-i}+\mu_{p-i}+\mu_{elas}$$

where *c_n_* is the molar concentration of water and *µ_n_^0^* is the chemical potential of pure water. The researchers defined four chemical potential terms (*µ_p‐n_, µ_n‐i_, µ_p‐i_*, and *µ_elas_*), which respectively represent interactions between polymer–water, water‐ions, polymer‐ions, and elasticity, as indicated by the subscripts.

According to basic thermodynamic principles, when a hydrogel reaches moisture absorption equilibrium with the environment, the chemical potential of water in the air (*µ_w,air_*) must equal the chemical potential of water within the hydrogel. The *µ_w,air_* can be expressed as:

(3) $$\mu_{w,air}=\mu_{w}^{0}+RTlnRH$$

where *R* represents the gas constant, *T* denotes the absolute temperature, and *RH* stands for the relative humidity. At this point, no net migration of water vapor occurs, and the moisture absorption capacity stabilizes at a constant value (equilibrium moisture absorption capacity).^[^ ^40^ ^]^

The thermodynamic behavior of hydrogel moisture absorption/desorption can be quantitatively described by two key parameters: moisture absorption capacity (an equilibrium quantity indicator) and adsorption enthalpy (an energy process indicator). In this study, these parameters were directly correlated through a thermodynamic model, jointly revealing the underlying mechanisms. The essence of the moisture absorption/desorption process lies in the shift of thermodynamic equilibrium between the “environment‐hydrogel” two‐phase system, driven by the difference in the chemical potential of water between the two phases. By combining the Equations above and conducting detailed derivations, researchers developed a predictive model for moisture absorption capacity:

(4) $$U=\frac{m_{w}}{m_{s}+m_{p}}=\frac{1}{1+1/SL}\cdot \frac{RH/\gamma_{w}\cdot i}{1-RH/\gamma_{w}}\cdot \frac{MW_{w}}{MW_{s}}$$

where *U* represents the moisture absorption capacity, *SL* denotes the salt loading (mass ratio of salt to polymer), *γ_w_* is the activity coefficient of water, *i* is the degree of dissociation of the salt (i.e., the number of ions dissociated per salt molecule, e.g., *i* = 2 for lithium chloride (LiCl)), and *MW_w_* and *MW_s_* are the molar masses of water and salt, respectively. This model indicates that the moisture absorption capacity of hydrogel‐salt composites is primarily determined by the type of salt, salt content, and RH, while the influence of the polymer is relatively minor.^[^ ^40^, ^43^ ^]^ Specifically, two salt‐related factors enhance moisture absorption capacity: first, a greater number of ions dissociated from the salt increases electrostatic attraction to water molecules; second, stronger ion‐water adsorption capacity lowers the chemical potential of water in the hydrogel. Both factors collectively contribute to higher moisture absorption. Additionally, higher salt loading increases ion concentration in the hydrogel network—providing more adsorption sites for water molecules and reducing the chemical potential of water within the hydrogel. This dual effect significantly enhances the equilibrium moisture absorption capacity.^[^ ^44^ ^]^ Furthermore, RH influences moisture absorption by altering the chemical potential of water in the air (*µ_vapor_*). Higher RH corresponds to a higher *µ_vapor_*, allowing the hydrogel to accommodate more water molecules at equilibrium, manifested as a nonlinear increase in equilibrium moisture absorption capacity with rising RH.

Adsorption enthalpy (*h_sor_*) is a key parameter that quantifies energy changes during the moisture absorption process. The adsorption enthalpy was derived as follows:

(5) $$h_{sor}=h_{lv}+h_{p-w}+h_{p-i}+h_{w-i}\approx h_{lv}+h_{w-i}$$

where *h_lv_* is the vaporization enthalpy of water, *h_pn_*, *h_pi_*, and *h_ni_* are enthalpy terms corresponding to interactions between polymer‐water, polymer‐ions, and water‐ions, respectively.​ This Equation indicates that the mixing enthalpy generated by water‐ion interactions (*h_ni_*) is directly related to the activity coefficient of water (*γ_n_)* via the Equation *h_ni_* = ‐RT ln*γ_n_*. Notably, *h_pn_* (polymer‐water) and *h_pi_* (polymer‐ion) contribute minimally to the total adsorption enthalpy—this is consistent with the parametric analysis of hydrogel‐salt composites, which confirmed that polymer‐related interactions have negligible effects on thermodynamic properties.^[^ ^40^ ^]^

The moisture absorption process involves two energy‐related steps. First, overcoming hydrogen bonds (*h_vap_*) between water molecules, which consumes energy (corresponding to *h_vap_* ≈2450 kJ kg^−1^ at 25  °C). Second, energy release through water‐ion interactions—a negative contribution since ion adsorption of water molecules is exothermic. The net adsorption enthalpy is the algebraic sum of these two components: the energy consumed to overcome *h_vap_* and the energy released by water‐ion interactions. Studies show that the adsorption enthalpy of hydrogels is ≈2800–2900 kJ kg^−1^ (at RH = 10%), only ≈20% higher than *h_vap_*. This relatively small increase indicates that the energy released by water‐ion interactions partially offsets the energy required for water vaporization, resulting in low net energy consumption during moisture absorption—the thermodynamic basis for hydrogels “low energy consumption” in atmospheric water harvesting. Beyond this, adsorption enthalpy exhibits dependence on RH. As RH increases, more water molecules are absorbed into the hydrogel, diluting salt concentration and reducing ion density.^[^ ^40^, ^45^ ^]^ This decrease weakens electrostatic attraction between ions and water molecules, causing the adsorption enthalpy to gradually approach *h_vap_*. This trend further verifies that water‐ion interactions are the core controlling factor for adsorption enthalpy.

#### 2.1.2 Factors Influencing Thermodynamic Changes of HPGs

Moisture sorption refers to the process in where water molecules migrate from the gaseous state (atmospheric water vapor) to the adsorbed state and bind to the material. During this process, water molecules combine with active sites on the material's surface or interior through hydrogen bonds, van der Waals forces, or chemical bonds, accompanied by heat release. The essence of this phenomenon lies in the transformation of water molecules from a “high‐energy free gaseous state” to a “low‐energy bound adsorbed state”, leading to a decrease in the total energy of the system. In contrast, desorption is the reverse process of sorption‐adsorbed water molecules break free from the interior of the material, convert back to the gaseous state, and are released into the environment. This process requires overcoming the binding forces (such as hydrogen bonds and chemical bonds) between water molecules and the material. Under normal temperature and natural conditions (without external intervention), the environment lacks sufficient energy to drive this process. Consequently, desorption rarely proceeds spontaneously.^[^ ^41^, ^46^ ^]^ This is precisely the reason why traditional hygroscopic materials need to be “heated at high temperatures” to supply energy for regeneration.

The moisture absorption process of HPGs is primarily regulated by two categories of factors: intrinsic material properties and external conditions/stimuli, both of which directly affect changes in chemical potential and Gibbs free energy. Different polymer network structures and chemical compositions lead to distinct water‐polymer interactions and transport properties. Crosslink density is a crucial parameter affecting the moisture absorption performance of hydrogels. A higher crosslink density results in a greater elastic modulus of the polymer network, which restricts the diffusion of water molecules and the movement of polymer chains, thereby reducing moisture absorption capacity. Meanwhile, high crosslink density also enhances the interaction between water molecules and polymer chains, influencing the distribution of chemical potential and changes in Gibbs free energy.^[^ ^43^ ^]^ However, the restricted chain mobility often dominates, limiting the accessibility of these sites and leading to a net decrease in moisture absorption capacity.

The type and density of functional groups on polymer chains significantly influence moisture absorption. Polar groups, such as hydroxyl, carboxyl, and amino groups, enhance this process by forming hydrogen bonds with water molecules. This interaction strengthens the water‐polymer network binding and lowers the chemical potential of the water molecules.^[^ ^47^ ^]^ In contrast, non‐polar groups weaken such interactions and inhibit moisture absorption. Additionally, the microstructure of HPGs (e.g., porosity, pore size distribution, and connectivity) affects the transport of water molecules and the distribution of chemical potential. Studies have shown that hydrogels with interconnected pore structures typically exhibit higher moisture absorption rates and greater moisture absorption capacity.

Environmental conditions and external stimuli (such as electric fields, magnetic fields, and light irradiation) also exert significant influences on changes in chemical potential and Gibbs free energy during the moisture absorption process. Temperature acts through a dual mechanism. On one hand, an increase in temperature enhances molecular motion and weakens the interaction between water molecules and the polymer network, thereby increasing the chemical potential of water and inhibiting moisture absorption to a certain extent. On the other hand, high temperature also raises the saturated vapor pressure of water vapor. Under the same RH, the chemical potential of water vapor in the environment increases, which may in turn promote moisture absorption. RH directly determines the magnitude of the driving force. The higher the humidity, the greater the chemical potential of water vapor in the atmosphere, and the larger the chemical potential difference between atmospheric water vapor and the interior of the HPGs. Figure 2D shows a schematic of the thermally‐driven desorption process in a thin hydrogel layer. During desorption, heat supplied to the hydrogel is conducted through the HPGs until it reaches the liquid‐gas interface of the hydrogel's micropores, thereby inducing water desorption. Subsequently, water vapor is released into the micropores and diffuses into the surrounding environment, driven by the vapor pressure difference between the hot liquid–gas interface and the ambient. As water continues to desorb, the local water concentration decreases while the polymer concentration increases, promoting diffusive transport of liquid from regions of high concentration to low concentration. Therefore, the ability of hydrogels to capture or release moisture depends on temperature, ambient RH, and the intrinsic properties of the sorbent.^[^ ^42^ ^]^

Beyond conventional environmental factors, external stimuli offer a new way to actively regulate moisture absorption behavior. For example, an electric field can alter the orientation and distribution of polar water molecules, affect their interaction with the polymer, adjust chemical potential distribution and Gibbs free energy changes, and thus significantly modify the moisture absorption rate and equilibrium capacity. Light irradiation primarily functions through two pathways: photothermal and photochemical effects.^[^ ^48^ ^]^ Photothermal materials can generate local heat under light irradiation, which indirectly affects moisture absorption through the temperature‐chemical potential‐free energy coupling mechanism. Photochemical reactions, on the other hand, can directly alter the hydrophilicity or crosslinking structure of the polymer network, enabling on‐demand regulation of the thermodynamic state.

### 2.2 Kinetics Mechanism of HPGs

#### 2.2.1 Theoretical Kinetics Model of HPGs

Hygroscopic kinetics is a fundamental aspect governing the performance of HPGs, encompassing the pathways, mechanisms, rates, and microscopic dynamics of water transport within their 3D networks. It is distinct from hygroscopic thermodynamics, which deals with equilibrium water uptake—where kinetics describes the rate of moisture sorption/desorption, thermodynamics determines the maximum capacity. Together, they provide a comprehensive framework for evaluating gel performance. A fundamental model in studying hygroscopic kinetics is Fick's law of diffusion, which is often applied to quantify the moisture sorption and desorption diffusion coefficients (denoted as *D*_ms_ and *D*_wd_, respectively). These parameters reflect how quickly water is absorbed or released by the gel, serving as key measures of its responsiveness and cycling efficiency. Their calculation Equations are as follows:

(6) $$\frac{M_{t}}{M_{e}}=\frac{2}{d}\sqrt{\frac{D_{ms}t}{\pi }}\left(0<\frac{M_{t}}{M_{e}}<0.6\right)$$

(7) $$\frac{\left(X_{t}-X_{e}\right)}{\left(X_{0}-X_{e}\right)}=1-\frac{2}{d}\sqrt{\frac{D_{wd}t}{\pi }}\left(0.4<\frac{\left(X_{t}-X_{e}\right)}{\left(X_{0}-X_{e}\right)}<1.0\right)$$

where, 𝑀_t_ and 𝑀_e_ represent the water content of the hydrated gel at time and under moisture absorption equilibrium, respectively, and d denotes the thickness of the dry gel. By plotting the curve of normalized weight (𝑀_t_/𝑀_e_) against the square root of time (*t* ^1/2^), the corresponding moisture sorption diffusion coefficient (𝐷_ms_) value of the gel can be obtained. *X*_0_, *X*_t_, *X*_e_ represent the water content of the hydrated gel, the gel at time *t*, and the gel under desorption equilibrium, respectively, 𝑑 is the thickness of the hydrated gel. By plotting the curve of normalized weight (*X*_t_‐X_e_)/(*X*_0_‐*X*_e_) against the square root of time (*t* ^1/2^), the corresponding desorption diffusion coefficient (𝐷_wd_) value of the gel can be obtained.^[^ ^49^ ^]^

As a classic theory describing the diffusion behavior of substances, Fick's Law occupies a fundamental position in the study of the dynamics of moisture‐absorbing gels and can be used to construct basic dynamic models to predict the moisture absorption/desorption behavior of gels. For example, for homogeneous gels such as pure gels, their 3D networks are uniform, and water diffusion paths are single. Based on Fick's Second Law, a diffusion model can be established to predict the water absorption capacity/water desorption capacity at different time points. Alternatively, in the stage before moisture absorption/desorption reaches equilibrium, if there is no significant change in the gel network structure (e.g., no severe pore shrinkage/expansion or component crystallization), even if the gel undergoes slight swelling, as long as the swelling rate is much slower than the diffusion rate, Fick's Law can still be used to approximately simulate the water migration process and quickly obtain key kinetic parameters. However, the mass transfer process in actual moisture‐absorbing hydrogel systems is often more complex, and the traditional Fick's Law cannot fully and accurately describe the water transport mechanism within hydrogels. For instance, when the hydrogel undergoes significant swelling, or when there are strong interactions between water and the hydrogel network (such as chemical adsorption, hydrogen bond association, etc.), water diffusion is no longer solely dominated by the concentration gradient, and its transport mechanism exhibits the complexity of multi‐factor coupling.

**Figure 3:**
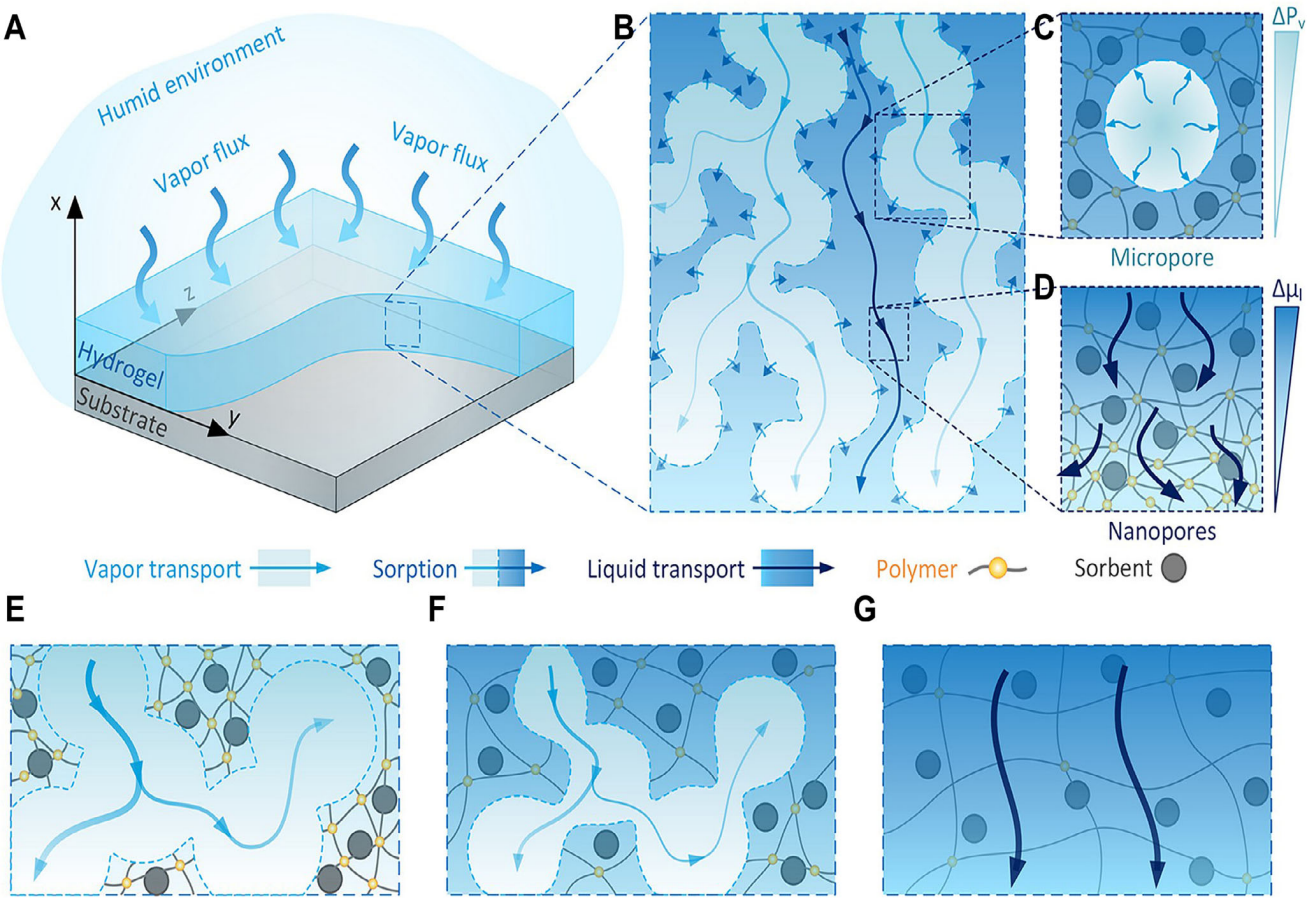
Sorption kinetics in HPGs. A) Schematic of a thin hydrogel layer absorbing moisture under humid conditions. B) Complex mass transport pathways within the hydrogel: vapor diffusion through micropores (light blue arrows), liquid transport in nanopores of the polymer network (dark blue arrows), and sorption at gas‐liquid interfaces (blue dashed lines). C) Enlarged view of vapor diffusion within a single micropore. D) Liquid transport through nanopores in the polymer matrix. E–G) Temporal evolution of hydration: initial vapor‐dominated transport through micropores E), intermediate stage with micropore shrinkage and polymer swelling F), and final homogeneous liquid diffusion upon full expansion G). Reproduced with permission.^[^ ^50^ ^]^ Copyright 2022, American Chemical Society.

In response to the complex mass transfer scenario where water vapor and liquid water coexist, the Two‐Concentration Model (TCM) achieves a more accurate characterization of the moisture absorption process, coupling the adsorption/desorption kinetics at the gas‐liquid interface. The “two concentrations” in TCM specifically refer to the molar vapor concentration of gaseous water molecules (*C*) and the molar concentration of condensed liquid water (*n*).^[^ ^50^ ^]^ Within the framework of this model, the liquid transport in hydrogels no longer relies on a single concentration gradient but is dominated by the water chemical potential gradient, and this driving mechanism plays a crucial role in rapid moisture absorption kinetics. Specifically, the mass transfer process in the system can be decomposed into three core links: Vapor Transport‐water vapor diffuses through the interconnected gaseous micropores inside the hydrogel, with the vapor pressure difference (*ΔP_v_*) as the driving force—meaning vapor spontaneously migrates from the region of higher pressure to the region of lower pressure (**Figure** **3**A–C). Liquid Transport‐liquid water is transported through the nanopores of the polymer network, driven by the water chemical potential difference (*Δµ_l_*) between the wet and dry regions of the network, which promotes the movement of liquid from the region of higher chemical potential to the region of lower chemical potential (Figure 3D). Interphase Coupling‐adsorption occurs at the gas‐liquid interface of the micropores, where water vapor is adsorbed and converted into liquid water (Figure 3E–G).^[^ ^50^ ^]^ This process tightly couples vapor transport and liquid transport, jointly determining the overall water transport efficiency and adsorption kinetic behavior within the HPGs.​ The TCM model's core structure comprises two governing equations—one for vapor transport and one for liquid water transport—as follows Equations:

(8) $$\frac{\partial \left(\emptyset_{g}C\right)}{\partial t}-\frac{\partial }{\partial_{x}}\left(D_{eff}\frac{\partial C}{\partial x}\right)=NSD_{V}\left(\frac{a_{w,s}P_{sat}}{RT}-C\right)$$

(9) $$\left(1-\emptyset_{g0}\right)\frac{\partial n}{\partial t}-\frac{\partial }{\partial x}\left(D_{l}\frac{\partial n}{\partial x}\right)=-NSD_{V}\left(\frac{a_{w,s}P_{sat}}{RT}-C\right)$$

where *C* is the molar concentration of water vapor in air, n is the molar concentration of liquid water in the hydrogel, *ϕ*_g_ is the gas‐phase volume fraction, *D*_eff_ and *D_l_*  are the effective diffusion coefficients of water vapor and liquid water, *N* is the micropore number density, *S* is the mass transfer shape factor of a single micropore, *D*_v_ is the diffusion coefficient of water vapor in air, *a*_w,s_ is the water activity of the salt solution, *P*_sat_ is the saturated vapor pressure of water, *R* is the gas constant, *T* is the absolute temperature.^[^ ^51^ ^]^ Equation (8) describes vapor transport along the hydrogel's thickness direction over time: the left side represents the change in vapor concentration and its gradient due to diffusion, while the right side corresponds to the interfacial adsorption/desorption term, which reflects the rate of condensation or evaporation at the gas–liquid interface. Equation (9) describes liquid water transport: its left side captures the temporal change in liquid water concentration and its diffusion‐driven gradient, and the right side is the inverse of the adsorption/desorption term from Equation (8). The TCM model comprises these governing equations for vapor and liquid water transport. It is especially suitable for highly porous hydrogels with coexisting multiphase water—such as freeze‐dried cellulose‐based hydrogels—as it quantifies how the chemical potential gradient drives liquid water transport. By revealing underlying mass transfer mechanisms, the TCM model offers a universal theoretical framework for understanding hydrogel dynamics and supports the design and optimization of adsorption‐based applications. For instance, in atmospheric water harvesting, passive cooling, and thermal management, the model facilitates in‐depth analysis of moisture sorption–desorption dynamics, enabling targeted improvements in hydrogel microstructure (e.g., porosity, pore size distribution) and composition to enhance system performance.^[^ ^41^ ^]^

#### 2.2.2 Key Factors Affecting the Moisture Sorption Kinetics of HPGs

Based on the mass transfer mechanisms revealed by the TCM model, the moisture sorption kinetics of HPGs are mainly determined by their intrinsic properties and microscopic morphology, which can be analyzed by decomposing the sorption process into three consecutive stages. 1) Surface adsorption, where atmospheric water vapor rapidly adsorbs onto active sites on the hydrogel surface. 2) Internal diffusion, during which adsorbed water molecules overcome mass transfer resistance and migrate into the internal pores and polymer chain interstices of the hydrogel. 3) Chain relaxation and swelling, where water interacts with polymer chains, prompting the transition of chain segments from a rigid to a flexible state and resulting in volumetric swelling of the hydrogel.^[^ ^52^ ^]^ Since the adsorption performance of hydrogels is inherently determined by their structural and property characteristics (as reflected in the above mechanism), the subsequent discussion focuses primarily on two core aspects: the intrinsic properties and microscopic morphology of HPGs.

**Figure 4:**
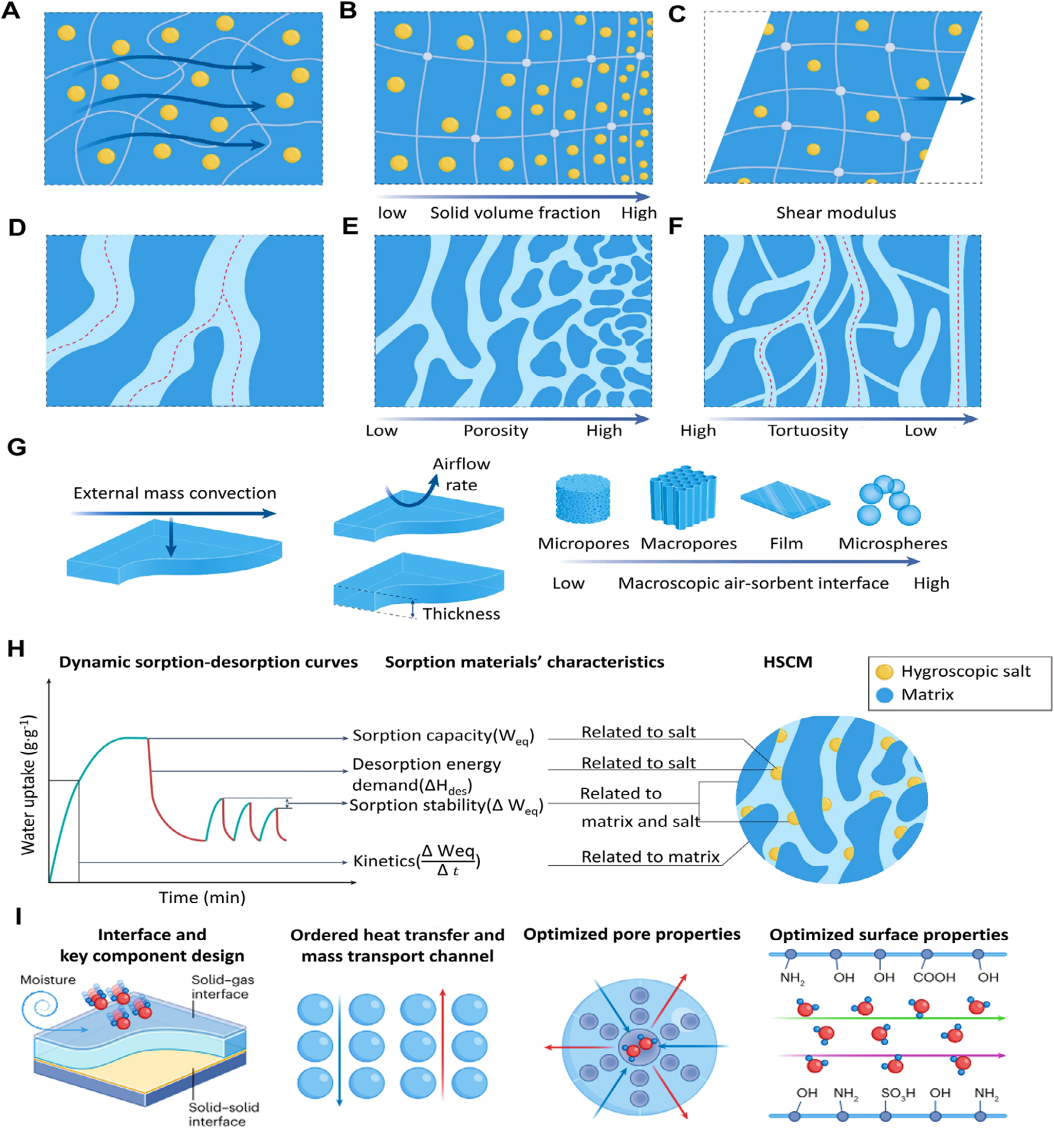
Key Factors Influencing Hygroscopic Kinetics in HPGs. A) Water transport mechanism in HPGs, regulated by solid volume fraction B) and shear modulus C). D) Vapor diffusion through matrix micropores, influenced by porosity E) and tortuosity F). G) External convective mass transfer, controlled by airflow rate, sorbent‐air contact area, and layer thickness per unit volume. H) Design framework illustrating the synergistic role of hygroscopic salt and polymer matrix in balancing sorption capacity, kinetics, enthalpy, and cycling stability. Reproduced with permission.^[^ ^1^ ^]^ Copyright 2024, Springer Nature. I) Strategies to enhance sorption‐desorption kinetics through multi‐scale ordered structures for efficient energy and mass transfer. Reproduced with permission.^[^ ^41^ ^]^ Copyright 2024, Springer Nature.

**Intrinsic property of HPGs**. Chemical composition determines the strength of the interaction between the hydrogel and water, and directly affects the moisture absorption kinetic process by regulating the adsorption driving force and diffusion energy barrier. Among them, hydrophilic groups such as hydroxyl, carboxyl, and amino can bind to water molecules through hydrogen bonds; the higher the density of these groups, the faster the initial adsorption rate of the hydrogel (**Figure** **4**A). Besides, the liquid diffusion coefficient (*D*_liquid_) is directly related to the Poisson ratio (*ν*), the shear modulus (*G*) and the permeability of the nanoporous polymer network (*k*) (Equation (10)):

(10) $$D_{\;liquid}=\frac{2\left(1+\upsilon \right)Gk}{3\left(1-2\upsilon \right)\eta }$$

where *η* is the dynamic viscosity of water. Optimizing the initial solid volume fraction (Figure 4B), increasing the shear modulus (Figure 4B) and augmenting the salt content within the hydrogel could all accelerate sorption kinetics. These adjustments relate closely to the crosslinking point density in the hydrogel and the intrinsic properties of the monomers.^[^ ^3^ ^]^

In HPGs fabricated by physical foaming, numerous interconnected micron‐scale pores are formed within the gel matrix, significantly reducing the diffusion resistance of moisture. The key to regulating mass transfer in such systems lies in the synergistic optimization of pore connectivity and pore size distribution. The freeze‐drying method enables the construction of vertically or horizontally aligned channel structures within HPGs by controlling ice crystal growth direction, thereby addressing the issue of tortuous mass transfer pathways typical of conventional disordered structures.^[^ ^1^, ^3^ ^]^ For instance, Cheng et al.^[^ ^29^ ^]^ produced HPGs with uniquely vertically oriented channel architectures via freeze‐drying. These structures provide abundant active surfaces and continuous transport pathways, allowing the material to maintain high adsorption performance even under low‐humidity conditions. Moreover, by leveraging evaporative cooling during adsorption‐desorption cycles and utilizing photovoltaic waste heat to drive water molecule release, this approach simultaneously enhances the power generation efficiency of solar panels and enables efficient freshwater harvesting. Similarly, 3D printing technology allows precise customization of the microarchitecture of HPGs. It can be used to design specialized structures such as gradient channels and grid‐like networks according to the mass transfer requirements of different application scenarios. This capability effectively overcomes the limitation of single‐path mass transfer in traditional disordered structures from a functional adaptability perspective.^[^ ^53^ ^]^

The microstructure also serves as the “physical carrier” for water transport in HPGs. Its key parameters directly determine the magnitude of mass transfer resistance and the exposure degree of adsorption active sites, thereby influencing the hydrogel's moisture absorption kinetic performance (Figure 4D). Specifically, the vapour diffusion coefficient (*D_eff_*) of HPGs is correlates to the porosity (*ϕ*) and diffusion hindrance structure factor (*p*) of the matrix, *p* is dimensionless and used to quantify the degree of hindrance that non‐porous structures (such as skeletons and particle aggregates) inside materials impose on the diffusion path of water molecules. (Equation (11)).^[^ ^1^ ^]^

(11) $$D_{eff}\propto \frac{1}{1+p\left(1-\emptyset \right)}$$

High porosity enhances the HPGs’ specific surface area and water transport pathways, thereby reducing diffusion resistance (Figure 4E). However, excessively high porosity compromises the structural stability of the polymer network and may even cause hydrogel collapse, which adversely affects long‐term moisture absorption performance.

To address this trade‐off, reducing the path length for internal vapor transport—particularly by decreasing pore tortuosity—can mitigate the negative impact of high porosity on mechanical properties while improving the effective diffusion coefficient (Figure 4F). For instance, freeze‐casting enables the fabrication of continuously ordered pore structures with specific orientations (e.g., lamellar, honeycomb, cellular, or radial patterns), which directly contributes to tortuosity reduction. The scale and uniformity of these ordered structures can be precisely tailored by controlling thermal gradients during freeze‐casting: unidirectional, bidirectional, or radial thermal gradients yield aligned lamellar arrays, large single‐domain structures, or radial pores, respectively. Furthermore, multi‐directional thermal gradients can guide pore formation along multiple preferred axes, thereby further shortening vapor diffusion distances. In addition to ordered pore structures, a hierarchical pore structure (e.g., micro‐meso‐macroporous networks) can synergistically facilitate efficient vapor adsorption (via micro/mesopores) and rapid liquid water transport (via macropores), thus optimizing the overall moisture absorption kinetics.^[^ ^54^, ^55^, ^56^ ^]^ Importantly, well‐interconnected pores throughout the network also eliminate mass transfer dead zones, minimize transport barriers, and significantly enhance the overall moisture absorption rate.

**Microscopic morphology of HPGs**. Macroscopic morphology exerts a flexible regulatory effect on moisture absorption kinetics by changing the contact area between the hydrogel and water, as well as the length of mass transfer paths (Figure 4G). Thickness is a key factor affecting kinetic performance: as the thickness increases, mass transfer paths extend accordingly, and the moisture absorption rate decreases exponentially. In addition, film‐like and fibrous hydrogels exhibit significantly better kinetic performance than bulk hydrogels, owing to their large specific surface area and short mass transfer paths. Sponge‐like hydrogels, which combine high specific surface area and a 3D connected structure, can simultaneously meet the requirements of “rapid adsorption” and “efficient transport”, making them the mainstream morphology in the field of atmospheric water harvesting.

In summary, the moisture behavior of HPGs is jointly determined by thermodynamics (equilibrium moisture capacity, direction of driving force) and kinetics (rate, transport mechanism). Thermodynamic properties—such as moisture absorption enthalpy and water chemical potential gradient—dictate the equilibrium moisture capacity and drive spontaneous absorption. Kinetic processes—including water diffusion and polymer chain relaxation—govern moisture adsorption/desorption rates and practical response efficiency (Figure 4H).^[^ ^1^ ^]^ Ideal HPGs design should combine high equilibrium moisture capacity with optimized kinetics, low enthalpy, and robust cyclic stability. This requires synergistic material engineering involving hydrophilic components, polymer network architecture, pore structure, and stimuli‐responsive functionality. Precise tuning of pore characteristics (size, distribution, connectivity), spatial arrangement of hydrophilic groups, and polymer chain dynamics enables systematic control over sorption capacity, rate, efficiency, and long‐term stability (Figure 4I).^[^ ^41^ ^]^

## 3 Classifications

Based on their moisture adsorption mechanisms, HPGs can be classified into two primary categories: static and responsive systems.^[^ ^42^ ^]^ Static HPGs capture water molecules mainly through physisorption or chemisorption via immobilized functional groups—such as hydroxyl, carboxyl, or ionic moieties—within a 3D polymer network. Their moisture uptake capacity remains largely independent of external stimuli such as temperature or light. In contrast, responsive HPGs incorporate dynamic functional components that enable programmable sorption/desorption switching. These systems achieve precise control over moisture capture and release through reversible conformational changes and cyclic network swelling–shrinking in response to external stimuli.

### 3.1 Static Network in HPGs

#### 3.1.1 Polyacrylic Acid (PAA)‐based HPGs

**Figure 5:**
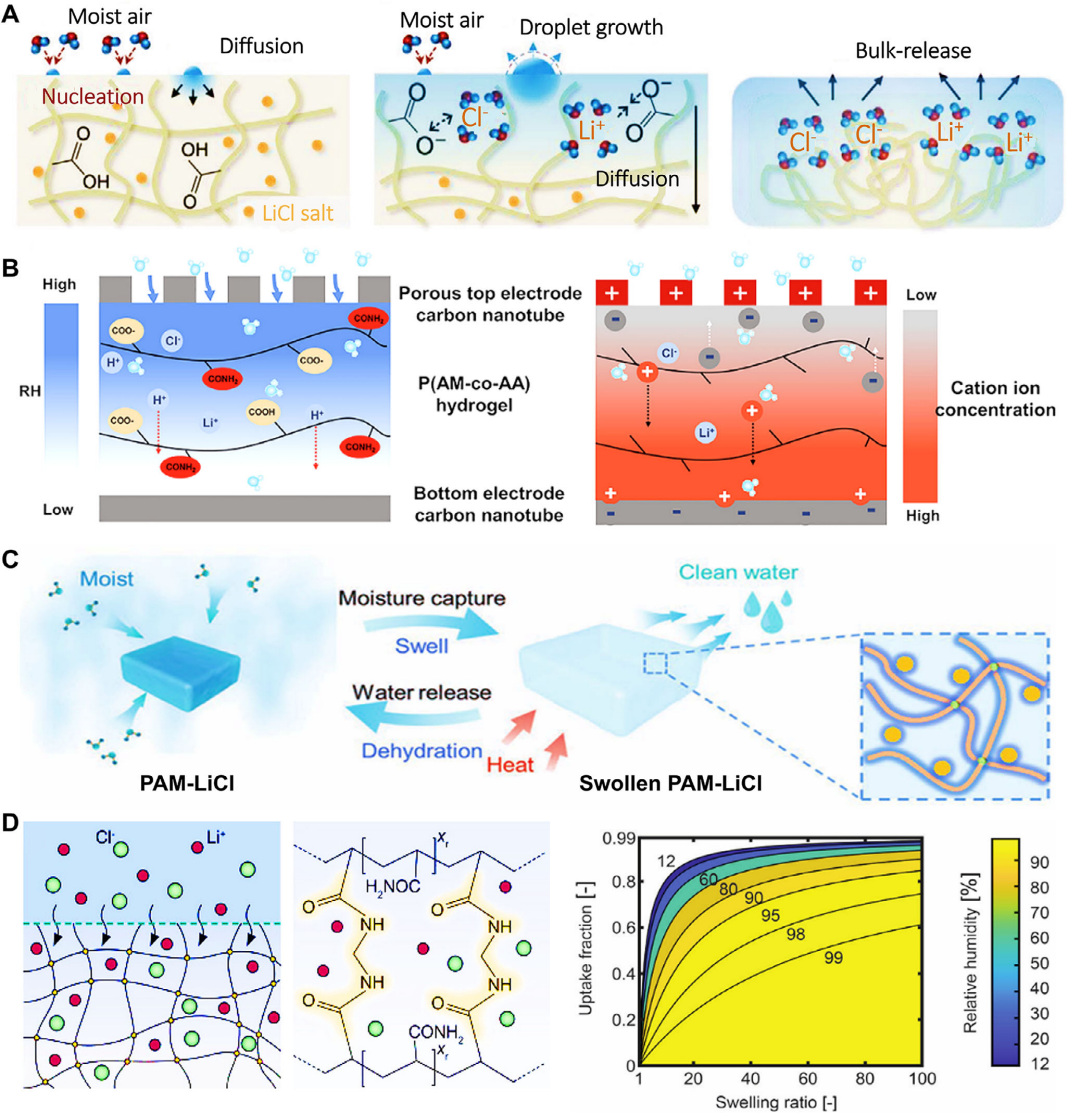
PAA and PAM Networks in HPGs. A) Moisture adsorption and spontaneous water release in a PAA‐LiCl film. Reproduced with permission.^[^ ^59^ ^]^ Copyright 2024, Wiley. B) Moisture adsorption process in a P(AM‐co‐AA) hydrogel. Reproduced with permission.^[^ ^60^ ^]^ Copyright 2024, Elsevier. C) Interfacial swelling between hydrogel and salt solution, with detailed gel network structure. Reproduced with permission.^[^ ^62^ ^]^ Copyright 2022, Wiley. D) AWH using LiCl and PAM‐LiCl, with modeling results of maximum allowable RH vsuptake fraction and swelling ratio. Reproduced with permission.^[^ ^63^ ^]^ Copyright 2024, Wiley.

PAA's molecular chains, densely functionalized with carboxyl functional (─COOH) groups, form strong hydrogen bonds with water molecules, resulting in significant hydration.^[^ ^57^ ^]^ In neutral/alkaline media, carboxyl ionization generates negatively charged chains, triggering electrostatic repulsion between anionic groups and thereby expanding the crosslinked network to enhance hygroscopic capacity. This dual mechanism of hydrogen bonding and electrostatic expansion grants PAA‐based materials superior hygroscopic performance.^[^ ^58^ ^]^ For instance, Yang et al.^[^ ^59^ ^]^ developed a lithium chloride LiCl‐doped PAA hydrogel that enables autonomous moisture sorption–desorption without external energy input (**Figure** **5**A). Its dual‐stage release mechanism—surface droplet nucleation driven by the disparity between nucleation and diffusion rates, and bulk water release from collapsed polymer chains—optimizes continuous water output. Additionally, PAA's carboxyl activity and tunable network structure enabled dual applications in moisture management and energy harvesting. For example, Qi et al.^[^ ^60^ ^]^ utilized a poly(acrylamide‐co‐acrylic acid) [P(AM‐co‐AA)] hydrogel to synergize electrical double layer (EDL) effects and humidity‐induced ion migration. Leveraging PAA's intrinsic properties, this copolymer enhanced ambient moisture‐enabled generators (MEGs) by exploiting EDL effects, yielding devices that maintain >0.8 V at 30% RH and >0.65 V over a temperature range of −10–60  °C (Figure 5B).

#### 3.1.2 Polyacrylamide (PAM)‐Based HPGs

Similar to PAA, PAM exhibits exceptional hydrophilicity, rapidly hydrating to form crosslinked networks. Combined with rapid kinetics, robust mechanical strength, tunable architecture, and broad humidity adaptability, these properties establish PAM as an ideal hydrogel matrix for moisture management.^[^ ^61^ ^]^ Exemplifying this, Yu et al.^[^ ^62^ ^]^ engineered a PAM‐LiCl hydrogel for rapid swelling kinetics, owing to hydrophilic chain‐water interactions that preferentially stabilize weakly bound/free water states, thereby reducing desorption enthalpy (Figure 5C). Consequently, the PAM‐LiCl hydrogel exhibited adsorption/desorption kinetics 2.3‐fold faster than the pure PAM hydrogels. Wang et al.^[^ ^63^ ^]^ proposed a PAM‐LiCl hydrogel for moisture harvesting in arid environments. By modeling the salt‐vapor equilibrium to optimize salt loading, the maximum leakage‐free RH was elucidated as a function of hydrogel water uptake and swelling ratio. These insights guide the design of HPGs with exceptional hygroscopicity (Figure 5D).

#### 3.1.3 Polyvinyl Alcohol (PVA)‐Based HPGs

PVA‐based HPGs inherently exhibit prominent hydrophilicity, a key attribute derived from the abundant hydroxyl groups in the PVA molecular backbone. Through precise control of physical or chemical crosslinking strategies, critical properties of these HPGs—including porosity, mechanical robustness, and swelling behavior—can be finely tailored. This structural tunability enables the optimization of moisture absorption performance, thereby expanding their applicability across diverse application scenarios.^[^ ^64^, ^65^ ^]^ For example, Tan et al.^[^ ^66^ ^]^ incorporated a zinc‐ethanolamine complex into a PVA matrix to create a zinc polyvinyl alcohol (Zn‐PVA) hydrogel. Leveraging strong molecular interactions with water, this material reduced internal humidity in protective clothing from 91% to 48.2%, alleviating heat stress for medical personnel. This strategy of functionalizing PVA matrices has also advanced electrochemical applications. Ren et al.^[^ ^35^ ^]^ developed a PVA‐based hydrogel that synergizes with high‐concentration potassium hydroxide to enhance ion mobility. Combining hygroscopicity with high ionic conductivity, it served as an effective electrolyte for supercapacitors under extreme humidity. Notably, further breakthroughs in PVA‐based hydrogel functionalization have been enabled by advanced interfacial engineering approaches. Recent studies demonstrated that partial coating of carbon black (CB) with a LiCl‐PVA hydrogel induces the formation of a self‐sustaining electric field (0.6–0.7 V) at the dry‐wet interface of the composite. The underlying mechanism relied on hygroscopic–ion energy conversion, mediated by electric double‐layer effects at the hydrogel‐CB interface. By precisely tailoring the deliquescence threshold of the LiCl‐PVA coating, predefined electrical responses can be triggered within specific humidity ranges—this unique feature enables innovative applications such as humidity‐responsive information encryption, highlighting the versatile functionality of PVA‐based HPGs.^[^ ^67^ ^]^

#### 3.1.4 Zwitterionic‐Based HPGs

**Figure 6:**
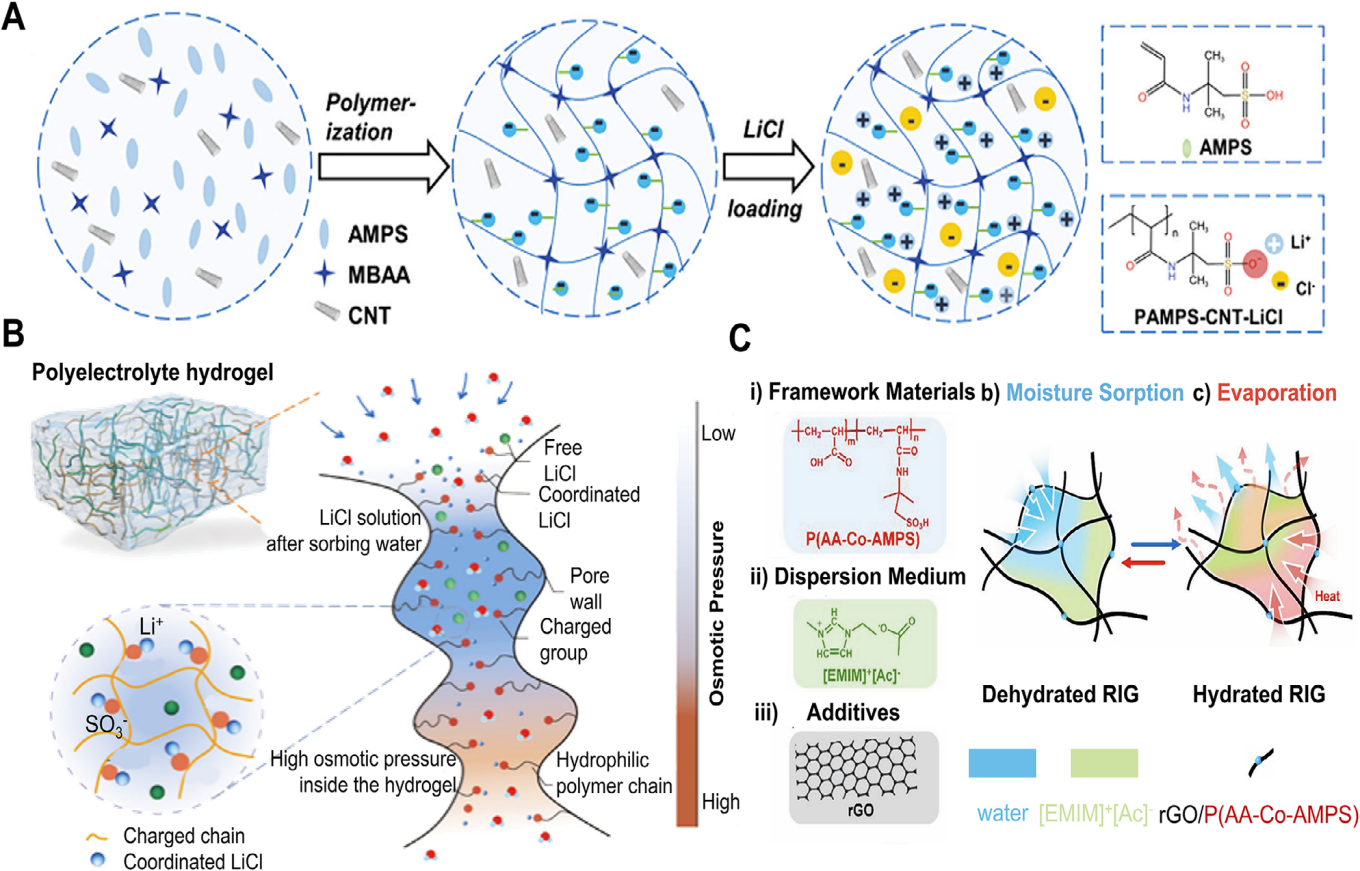
Zwitterionic Networks in HPGs. A)Preparation and characterization of the PAMPS‐CNT‐LiCl hydrogel sorbent, including the states of free and coordinated LiCl. B) Structure of a polyelectrolyte hydrogel with coordinated LiCl and oppositely charged hydrophilic polymer chains, illustrating its mechanism for efficient water sorption, transport, and storage. Reproduced with permission.^[^ ^71^ ^]^ Copyright 2023, Wiley. C) Structure and atmospheric moisture sorption behavior of dehydrated RIG. Reproduced with permission.^[^ ^72^ ^]^ Copyright 2021, Wiley.

Zwitterionic HPGs exhibit synergistic moisture sorption by forming robust hydration networks via electrostatic interactions, enabling high adsorption kinetics and sustained water retention simultaneously.^[^ ^68^ ^]^ The pH‐responsive nature of their charged functional groups further ensures stable performance across a wide range of humidity conditions, endowing the materials with exceptional environmental adaptability, high adsorption efficiency, and durable moisture retention.^[^ ^69^ ^]^ A critical mechanism underlying their high hygroscopic performance is the anti‐polyelectrolyte effect—a unique phenomenon where zwitterionic polymer chains expand rather than shrink in high‐ion environments (e.g., LiCl doping). For instance, Yu et al.^[^ ^70^ ^]^ incorporated LiCl into a poly(2‐(dimethylamino)ethyl methacrylate)‐grafted polysulfone (PDMAPS) hydrogel. By leveraging the anti‐polyelectrolyte effect—which promotes chain expansion in high‐ion environments, and the expanded polymer chain structure provides more sites for interaction with water molecules to facilitate water infiltration and adsorption—the system exhibited enhanced polymer solubility and swelling behavior, achieving a water vapor adsorption capacity of 0.62 g·g^−1^ at 30% RH within 120 min. Electrostatic interactions between the polymer and salt also contribute to remarkable long‐term stability. Beyond adsorption performance, Wang et al.^[^ ^71^ ^]^ enhanced the internal osmotic pressure of polyelectrolyte hydrogels and coupled it with a hybrid desorption strategy using solar energy and waste heat, enabling all‐weather rapid water release (**Figure** **6**A,B). The system maintained a water production rate of 2410 mL·kg^−1^·day^−1^ over eight consecutive cycles. Further structural optimization was achieved by Chen et al.^[^ ^72^ ^]^ who introduced the hygroscopic ionic liquid 1‐ethyl‐3‐methylimidazolium acetate ([EMIM]^+^[Ac]^−^) and reduced graphene oxide (rGO) to fabricate a solid rGO/ionogel (RIG) (Figure 6C). Featuring a highly entangled network and interconnected macroporous structure, the composite demonstrated rapid and high‐capacity moisture uptake, reaching 0.96, 0.50, and 0.24 g·g^−1^ after 12 h at 90%, 70%, and 50% RH, respectively.

#### 3.1.5 Biomass‐Derived HPGs

Biomass‐derived HPGs are 3D networks prepared via physical/chemical crosslinking or biosynthesis of natural biomass and its derivatives. They function through efficient atmospheric moisture capture and retention, governed by mechanisms including physical adsorption, hydrogen bonding, and electrostatic interactions.^[^ ^25^ ^]^ Benefiting from renewability, biodegradability, and structural tunability, these materials show great promise in humidity control, AWH, and moisture‐enabled energy conversion.^[^ ^73^ ^]^ In passive dehumidification, research emphasizes rapid moisture uptake and scalable humidity regulation.^[^ ^74^ ^]^ Chen et al.^[^ ^75^ ^]^ developed a nanostructured moisture‐absorbing gel (N‐MAG) consisting of a LiCl‐modified hydrophilic nanocellulose network. Its interconnected nanochannels facilitate rapid water transport, prevent surface accumulation, and enable ultrafast sorption. With high scalability, N‐MAG reduced ambient humidity from 96.7% to 28.7% within 6 h.

**Figure 7:**
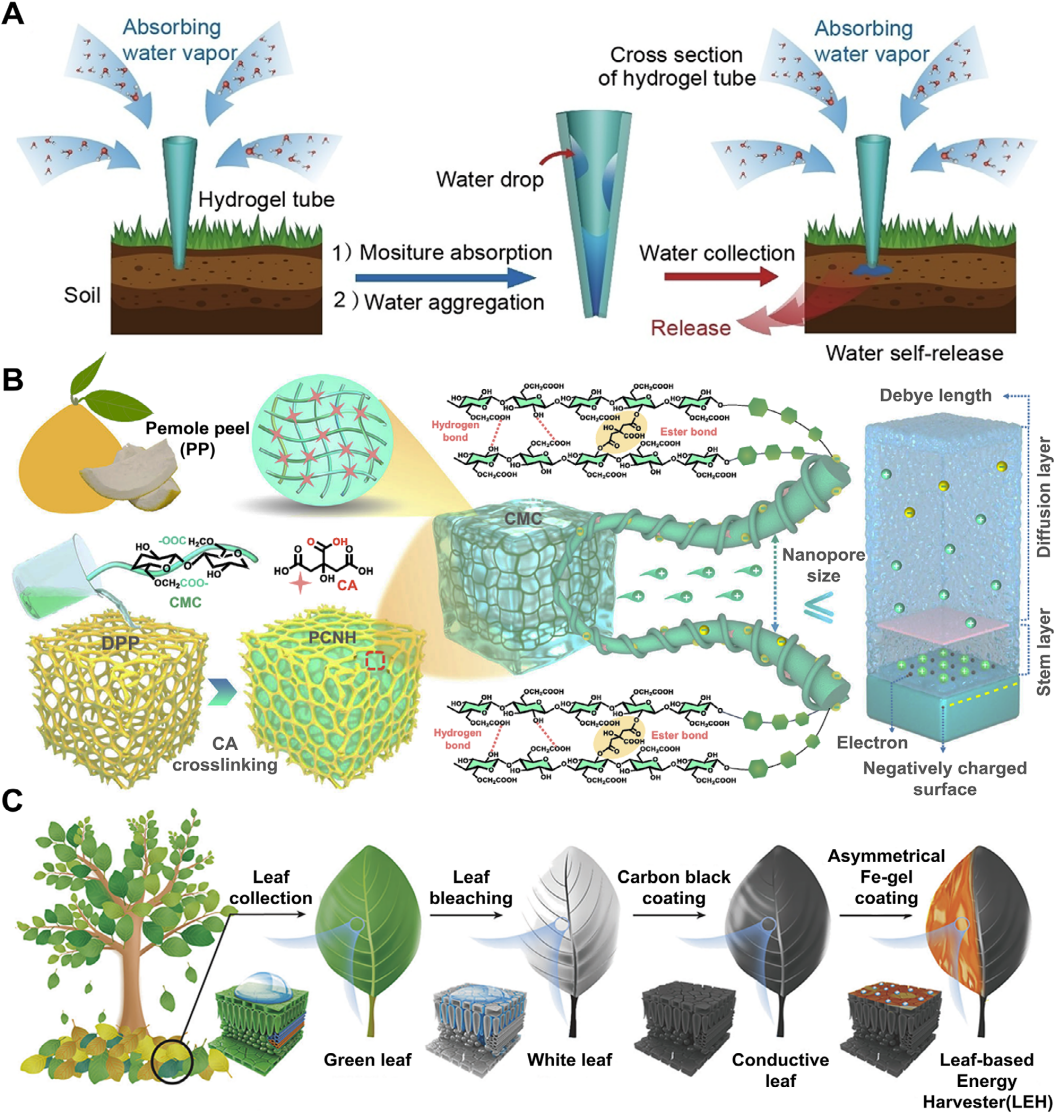
Biomass‐derived HPGs. A) Application of hydrogel tubes in plant irrigation systems. Reproduced with permission.^[^ ^76^ ^]^ Copyright 2024, Wiley. B) Preparation and nanopore structure of PCNH, showing enhanced cation selectivity due to strong electric double layer (EDL) overlap within sub‐Debye‐length nanopores. Reproduced with permission.^[^ ^77^ ^]^ Copyright 2025, Springer Nature. C) Structure and moisture sorption behavior of dehydrated reduced graphene oxide/ionogel (RIG). Reproduced with permission.^[^ ^78^ ^]^ Copyright 2025, Springer Nature.

For AWH applications, biomass HPGs aim to enhance adsorption/desorption kinetics and energy efficiency. Zhang et al.^[^ ^76^ ^]^ fabricated conical hollow hydrogel tubes through Ca^2+^‐crosslinked sodium alginate (SA), enabling spontaneous moisture capture and energy‐free release at room temperature, offering a potential water source for arid regions (**Figure** **7**A). Wang et al.^[^ ^79^ ^]^ developed an LiCl‐doped SA nanocomposite, which achieved a water uptake of 6.61 kg·kg^−1^ at 30% RH and a daily output of 2.8 L·kg^−1^ in solar‐driven AWH. Beyond humidity management, biomass HPGs also exhibited strengths in moist‐electric energy conversion. Qi et al.^[^ ^77^ ^]^ synthesized nanopore‐confined hydrogels (PCNH) using pomelo peel frameworks crosslinked with citric acid and carboxymethyl cellulose. The nanostructure enhances moisture adsorption, ion dissociation, and directional ion migration under the Debye screening effect, delivering an open‐circuit voltage of 1.51 V and a current density of 740.5 µA·cm^−2^ at 90% RH (Figure 7B). Tan et al.^[^ ^78^ ^]^ constructed a leaf‐enabled harvester (LEH) that utilizes the electric double layer (EDL) effect during water sorption. At 25 °C and 75% RH, LEH produced 49 µA·cm^−2^ and 497 µW·cm^−3^, with a self‐recharging capability that sustained operation for 240 h after discharge (Figure 7C).

These advancements in biomass HPGs performance stem from rational selection of biomass feedstocks and precise engineering of their microstructures. Key strategies driving performance optimization include enhancing interactions between hydrophilic functional groups and water molecules, as well as constructing hierarchical porous architectures to accelerate mass transfer.^[^ ^80^ ^]^ Collectively, these efforts have established biomass HPGs as versatile, eco‐friendly materials with broad prospects for multidisciplinary applications across humidity control, water harvesting, and energy conversion.

### 3.2 Dynamic Network HPGs

Traditional HPGs rely on static hydrophilic groups for passive moisture capture. However, their fixed functional group distribution and network structure lead to drawbacks such as humidity‐dependent sorption efficiency and high energy consumption for water release at elevated temperatures.^[^ ^81^, ^82^, ^83^ ^]^ In contrast, emerging dynamic‐network HPGs feature reversible crosslinking structures formed through dynamic chemical bonds or physical interactions. These materials offer two key advantages: first, the reversible bonds enable structural integrity over repeated sorption–desorption cycles, preventing disruption of charge balance caused by salt migration and aggregation—thereby sustaining long‐term hygroscopic performance.^[^ ^84^ ^]^ Second, they can adapt their network topology in response to external stimuli, enabling efficient moisture uptake and energy‐efficient water release.^[^ ^85^, ^86^ ^]^

#### 3.2.1 Temperature‐Responsive HPGs

**Figure 8:**
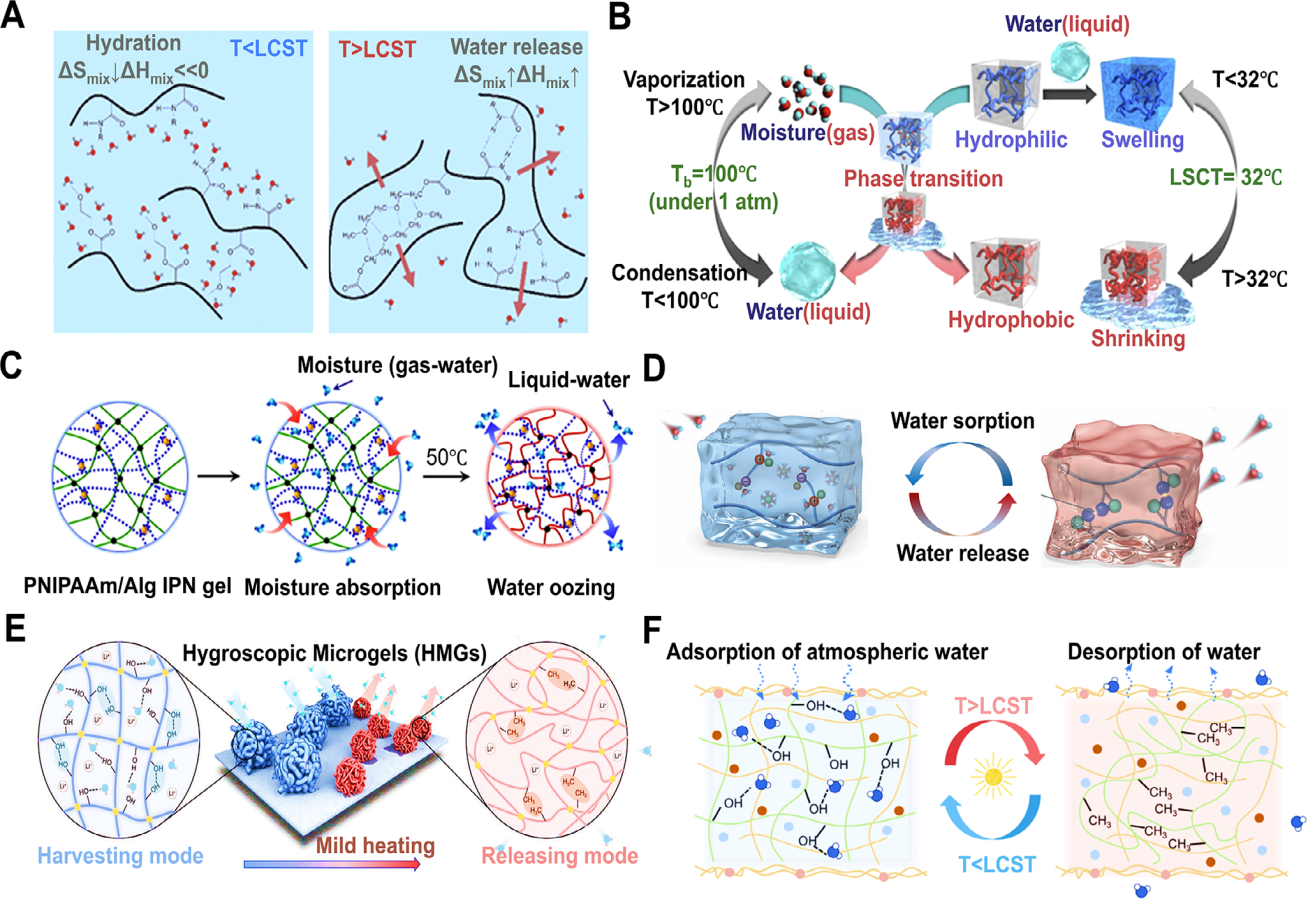
Dynamic Network HPGs. A) Phase behavior and molecular interactions during the LCST transition. B) Adsorption and oozing behavior of a dried PNIPAAm/alginate IPN gel. C) Moisture absorption and release in IPN gels induced by temperature changes. Reproduced with permission.^[^ ^89^ ^]^ Copyright 2018, Springer Nature. D) Hydrogel swelling at room temperature (sorption state) and contraction upon heating (release state), driven by hydrophobic interactions. Reproduced with permission.^[^ ^90^ ^]^ Copyright 2025, Wiley. E) Schematic of a CAL gel with photothermal conversion and rapid sorption‐desorption kinetics. Reproduced with permission.^[^ ^93^ ^]^ Copyright 2024, Wiley. F) Design of an HMG for enhanced AWH. Reproduced with permission.^[^ ^94^ ^]^ Copyright 2024, Wiley.

Poly(N‐isopropylacrylamide) (PNIPAm) hydrogel exhibits temperature‐responsive behavior ideal for regulating moisture sorption–desorption cycles.^[^ ^86^ ^]^ With a lower critical solution temperature (LCST), it operates via ambient temperature changes or low‐grade heat. Below the LCST, amide groups form strong hydrogen bonds (≈20–25 kJ·mol^−1^) with water, leading to chain expansion and hydration that enhance moisture uptake. Above the LCST, hydrophobic isopropyl interactions prevail, triggering chain collapse into micelles and expelling water through low‐energy desorption aided by pore closure (**Figure** **8**A).^[^ ^87^, ^88^ ^]^ Miyata et al.^[^ ^89^ ^]^ developed an interpenetrating network (IPN) gel that captures moisture below the LCST and releases liquid water above it (Figure 8B,C). Yu et al.^[^ ^27^ ^]^ created an ultra‐hygroscopic composite of polypyrrole chloride (PPy‐Cl) and PNIPAm, capable of absorbing atmospheric moisture multiple times its mass and releasing clean water under sunlight. Further advancing this approach, Yu et al.^[^ ^90^ ^]^ grafted thermoresponsive hydroxypropyl (HP) groups onto polysaccharides via alkylation. The HP groups disrupted the polysaccharide hydrogen network and impart thermoresponsive hydrophobicity, enabling efficient water release upon heating. The optimized hydrogel achieved adsorption capacities of 0.86–1.32 g·g^−1^ (15‐30% RH) and released 95% adsorbed water at 60 °C (Figure 8D). To overcome salting‐out, Yu et al.^[^ ^91^ ^]^ developed a thermoresponsive microgel (TZMG). This design confines hygroscopic sites (e.g., anchoring LiCl) to promote ordered hydration structures, preventing disordered liquefaction. Mild heating triggers conformational changes, disintegrating hydration networks for rapid water release and superior adsorption‐desorption kinetics. In parallel, Guo et al.^[^ ^92^ ^]^ designed a temperature‐sensitive interpenetrating hydrogel. Heating reversibly dissociates dynamic hydration structures, driving autonomous carbon nanotube migration to form a surface photothermal layer; cooling reverses this process. This material exhibits exceptional adsorption/desorption kinetics, achieving a water production rate of 2.57 L·kg^−1^·d^−1^ in continuous cycles—a 40% improvement over traditional materials.

#### 3.2.2 Thermo‐Responsiveness HPGs

Hydroxypropyl cellulose (HPC) hydrogels offer superior biocompatibility, salt tolerance, and cyclic durability over PNIPAm analogues. Their renewable architecture, tunable LCST (≈42 °C), and high‐density hydroxyl groups enable temperature‐triggered moisture regulation. Key to this behavior is the dynamic dissociation/recombination of HPC‐water hydrogen bonds, driving reversible chain expansion (below LCST) for moisture capture and contraction (above LCST) for water release.^[^ ^95^ ^]^ Harnessing this advantage, Chen et al.^[^ ^94^ ^]^ integrated SA, HPC, and demethylated lignin into a composite hydrogel. This material achieved adsorption and desorption rates of 1.41–1.74 and 1.98 kg·kg^−1^·h^−1^ at 15–30% RH (Figure 8E). Additionally, Yu et al.^[^ ^93^ ^]^ developed hygroscopic microgels (HMGs) leveraging short‐range diffusion and hydrophilic‐hydrophobic switching, enabling 24–36 daily cycles and a record water yield of 19.1 L·kg^−1^·d^−1^ (30% RH). This strategy presents a sustainable and efficient approach for designing HPGs with exceptional sorption kinetics (Figure 8F).

## 4 Construction Strategy of HPGs

### 4.1 Sol–Gel Engineered HPGs

The sol–gel method offers molecular‐level control over pore nanostructure and inorganic salt loading, enabling highly efficient moisture capture.^[^ ^96^ ^]^ In contrast to freeze‐drying or physical foaming, this technique allows precise regulation of pore size, morphology, and surface functional groups through modulation of sol–gel reaction kinetics. For instance, Nandakumar et al.^[^ ^26^ ^]^ developed superhygroscopic nanoporous hydrogels (SNHGs) featuring striated‐contour nanopores, which captured over 360 wt.% moisture at 90% RH within 12 h. Relying solely on physical adsorption, the SNHGs demonstrate remarkable cyclic stability and regeneration efficiency, retaining structural integrity and hygroscopic performance across repeated sorption‐desorption cycles.

### 4.2 Polymerization Crosslinking for HPGs

**Figure 9:**
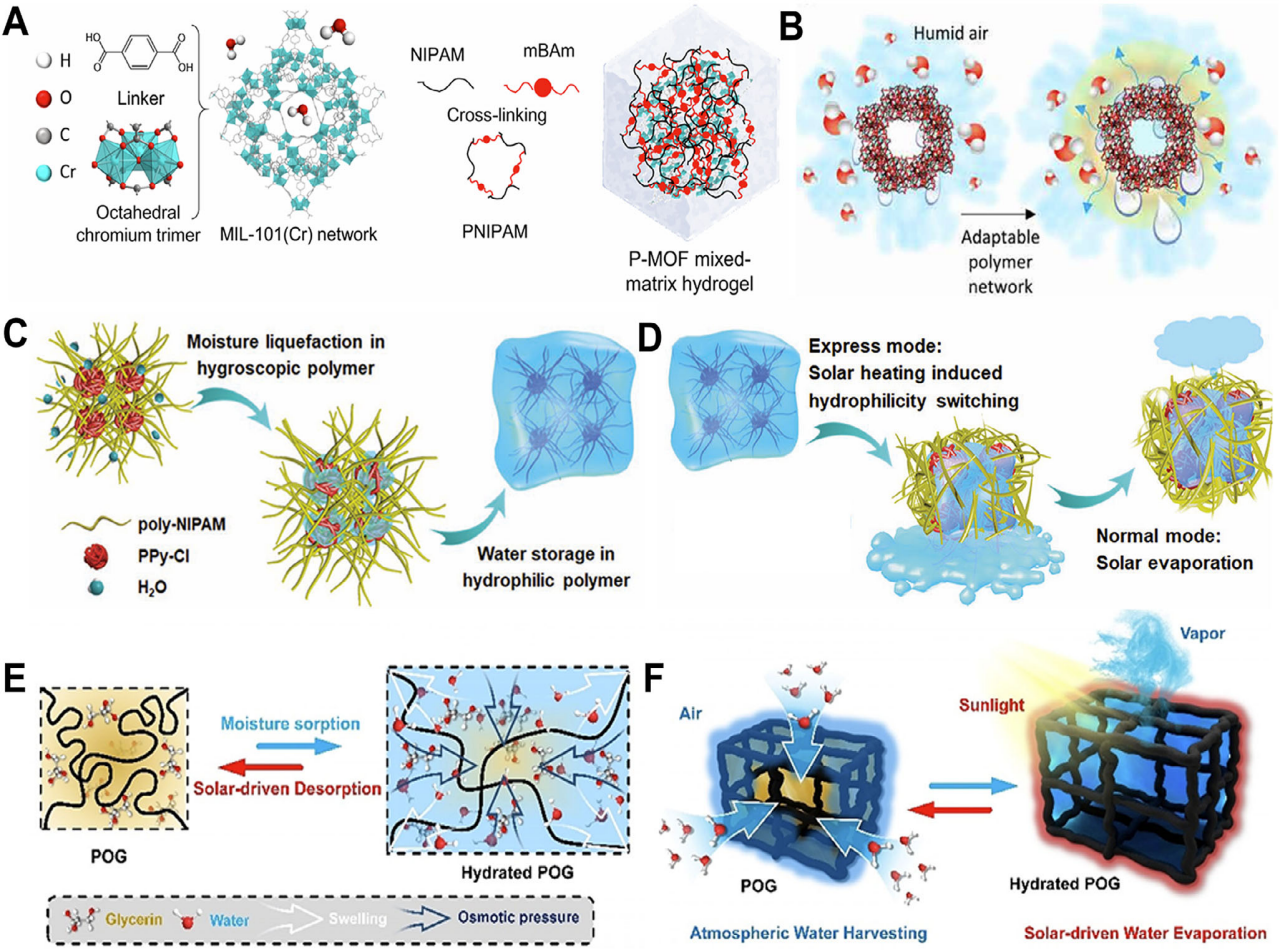
Chemical crosslinking strategy of HPGs. A) Crystal structure of MIL‐101(Cr), crosslinking of NIPAM with mBAm, and preparation of the P‐MOF mixed‐matrix hydrogel. B) Mechanism of photothermal water evaporation. Reproduced with permission.^[^ ^98^ ^]^ Copyright 2020, American Association for the Advancement of Science. C) Moisture adsorption process in SMAGs. D) Solar‐driven water release via express and standard modes. Reproduced with permission.^[^ ^27^ ^]^ Copyright 2019, Wiley. E) Moisture sorption mechanism in POG: capture, transport via osmotic pressure, and storage through swelling. F) Solar‐powered AWH using POG. Reproduced with permission.^[^ ^11^ ^]^ Copyright 2020, Wiley.

This method employs covalently bonded hydrophilic polymer skeletons that leverage synergistic effects between pore networks and hydrophilic groups to enhance water adsorption and retention.^[^ ^97^ ^]^ For instance, Ho et al.^[^ ^98^ ^]^ synthesized a polymer‐MOF (PC‐MOF) gel through in situ radical polymerization of highly hygroscopic MIL‐101(Cr) with NIPAM monomers. Subsequent salinization with calcium chloride (CaCl_2_) yielded a PC‐MOF material dually loaded with both inorganic salts and MOF. This system exhibited spontaneous water release, overcoming droplet coalescence through the combined effects of gravity and surface energy, thereby facilitating moisture management in humid environments (**Figure** **9**A,B). In another approach, Yan*g *et al.^[^ ^59^ ^]^ fabricated LiCl‐embedded PAA hydrogels via UV polymerization. At 50–90% RH, these hydrogels simultaneously captured moisture and achieve autonomous liquid water release by promoting droplet nucleation and growth through differential diffusion rates, while polymer chain stacking drives expulsion of water from the network.

Furthermore, constructing interpenetrating network (IPN) structures represents an effective strategy for enhancing hygroscopic performance. Yu et al.^[^ ^27^ ^]^ developed an IPN composed of PPy‐Cl and PNIPAM, resulting in a super moisture‐absorbent gel (SMAG) with a moisture adsorption capacity of 0.7–6.7 g·g^−1^ across 30–90% RH. The mechanism involves PPy‐Cl facilitating moisture adsorption and liquefaction into water clusters, which are then efficiently transported through the porous network (Figure 9C,D). Additionally, encapsulating hygroscopic liquids within gel networks via solvent replacement is another important method. For instance, Chen et al.^[^ ^11^ ^]^ prepared a composite hygroscopic organogel (POG) by replacing the solvent in a hydrophilic polymer network with glycerol. This design enables continuous, rapid, and high‐capacity moisture harvesting through synergistic interaction between the polymer framework and glycerol. The ion exchange method is also an effective strategy for preparing HPGs by selectively replacing free ions in gels (Figure 9E,F). Wu et al.^[^ ^99^ ^]^ synthesized a metal‐ and halide‐free HPGs through exchange with organic ions. In this system, both the charged gel backbone and paired organic anions contribute to moisture absorption, and the equilibrium water uptake can be precisely tuned by selecting appropriate counteranions. Recently, Shi et al.^[^ ^45^ ^]^ compounded LiCl with the polymer hydrogel matrix poly(acrylamide)‐2‐hydroxyethyl acrylate to prepare a moisture‐absorbing gel (PL). It enabled rapid moisture absorption via its hydrophilic functional groups. Meanwhile, its porous structure can stably retain the captured moisture inside, effectively suppressing leakage. The PL hydrogel. This material possessed both AWH performance with capacity of 4.49 g·g^−1^ and hydrogen production capability with a rate of 17.5 mmol·m^−2^·h^−1^.

### 4.3 Physical Foaming for HPGs

**Figure 10:**
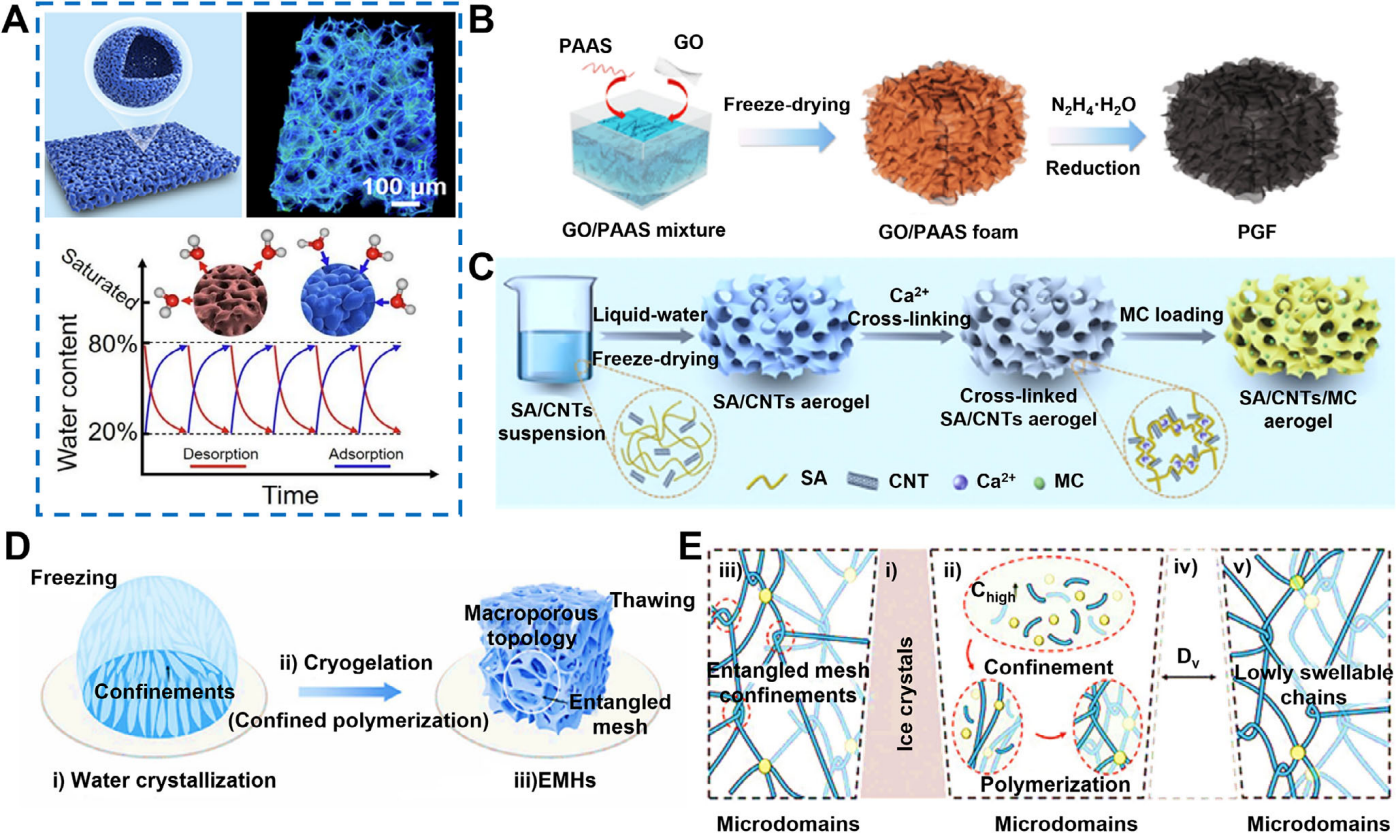
Physical foaming and Freeze‐drying for HPGs. A) Structural schematic, 3D X‐ray microscopy reconstruction, and "8020″ operational principle for continuous water capture of the THL system. Reproduced with permission.^[^ ^28^ ^]^ Copyright 2023, AIP Publishing. B) Preparation process of PGF. Reproduced with permission.^[^ ^101^ ^]^ Copyright 2020, Wiley. C) Fabrication procedure of SA/CNTs/MC aerogels. Reproduced with permission.^[^ ^102^ ^]^ Copyright 2024, Wiley. D) Macroporous topology of EMHs achieved via cryogelation to enhance mass transport. E) Schematic of the cryogelation process. Reproduced with permission.^[^ ^103^ ^]^ Copyright 2024, Wiley.

This approach constructs hydrophilic polymer networks through covalent crosslinking, leveraging the synergistic effects of porous architectures and hydrophilic functional groups to enhance water adsorption and retention.^[^ ^100^ ^]^ For example, Wang et al.^[^ ^28^ ^]^ employed hydroxypropyl methylcellulose and lithium chloride within this framework to fabricate hollow spherical porous gels (≈200 µm). The highly porous structure endowed the material with exceptional moisture uptake, achieving adsorption capacities of 1.18 and 2.86 g·g^−1^ at 15% and 60% RH, respectively, along with excellent cyclic stability. Based on this material, the authors developed a water harvesting prototype with parallel desorption chambers and condensers, which demonstrated high water production rates of 3.82 L·kg^−1^·d^−1^ in summer and 2.98 L·kg^−1^·d^−1^ in winter (**Figure** **10**A). Further advancing this strategy, Wang et al.^[^ ^83^ ^]^ engineered a HPG featuring both high porosity and high pore connectivity via physical foaming. This hierarchical structure significantly reduced vapor diffusion resistance, leading to markedly enhanced adsorption–desorption kinetics. The material also exhibited high equilibrium uptake capacities of 1.01, 2.03, and 6.83 g·g^−1^ at 30%, 60%, and 90% RH, respectively. Importantly, the combination of controlled polymer swelling and strong capillary forces within the porous matrix effectively prevented salt leakage, ensuring outstanding structural and functional stability over multiple cycles even under high humidity.

### 4.4 Freeze‐drying for HPGs

The freeze‐drying technique utilizes a unique ice‐templating mechanism that preserves pore structures by controlling the growth direction and size of ice crystals, thereby enabling precise design of pore orientation and size distribution in HPGs.^[^ ^54^ ^]^ This approach enhances the synergy between physical and chemical adsorption, while also improving the structural stability, moisture absorption performance, and functional properties of HPGs.^[^ ^100^ ^]^ For instance, Qu et al.^[^ ^101^ ^]^ developed a poly(sodium acrylate)/graphene framework gel (PGF), which combined the hydrogen‐bond capture ability of carboxyl groups on polyacrylic acid sodium salt (PAAS) chains with efficient mass transfer through porous channels, achieving an adsorption capacity of 5.20 g·g^−1^ and rapid hygroscopic kinetics at 100% RH (Figure 10B). Tan et al.^[^ ^102^ ^]^ fabricated a sodium alginate/carbon nanotubes/microcrystalline cellulose (SA/CNTs/MC) aerogel featuring hierarchical pores formed via unsaturated coordination between hygroscopic salts and organic ligands, significantly enhancing water transport rates. This material exhibited high water uptake capacities of 5.43 and 0.27 kg·kg^−1^ at 95% and 20% RH, respectively (Figure 10C). Chen et al.^[^ ^103^ ^]^ constructed entangled network hydrogels (EMHs) via freeze‐drying. The microdomain structure formed by solute aggregation endows ultrafast hygroscopic kinetics of 1.08 g·g^−1^ at 30% RH (Figure 10D,E).

**Figure 11:**
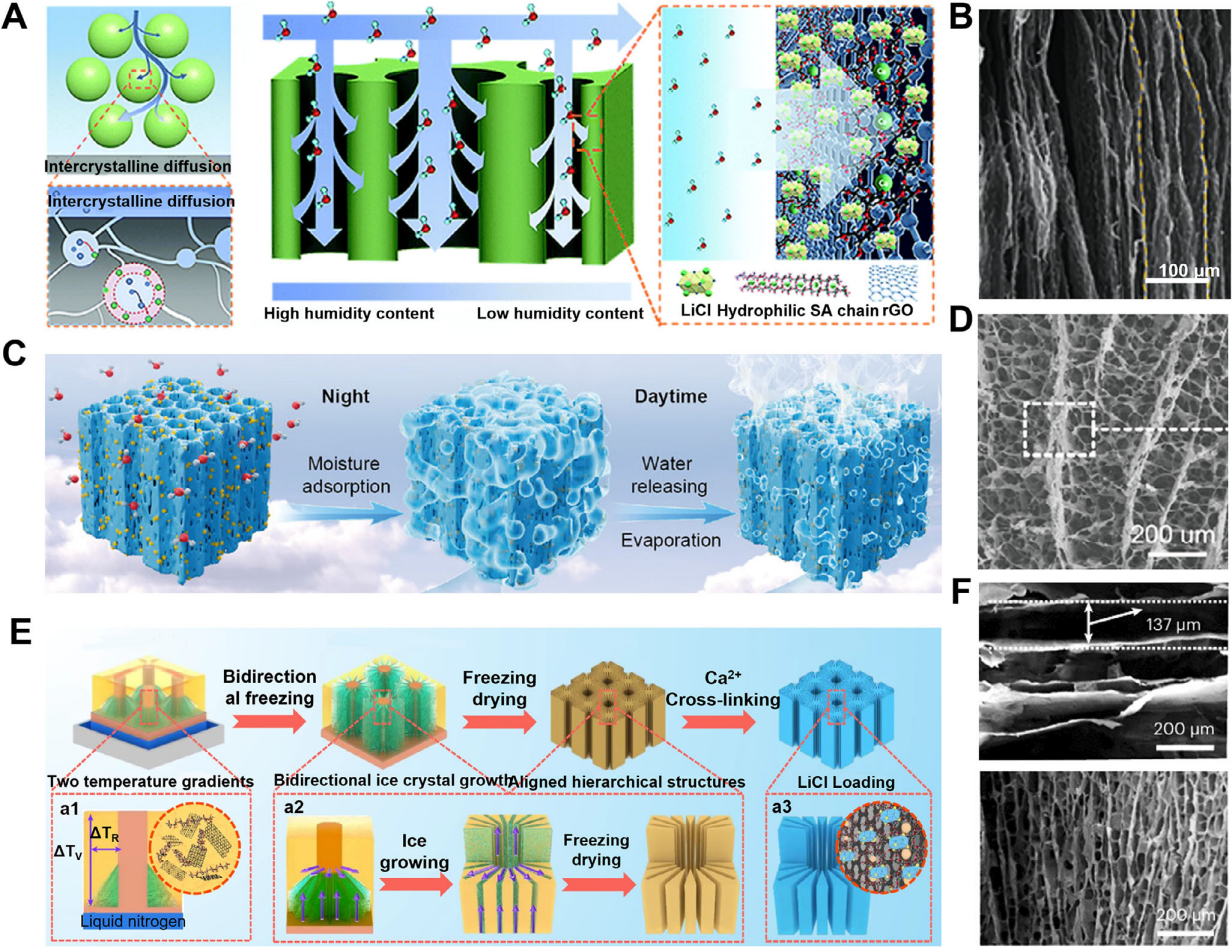
Freeze–drying for HPGs. A,B) Schematic diagram comparing water vapor transport paths from ambient air to randomly arranged traditional packed adsorbents and vertically aligned nanocomposite adsorbents and its corresponding SEM image showing the vertically aligned structure. Reproduced with permission.^[^ ^106^ ^]^ Copyright 2021, Royal Society of Chemistry. C,D) Schematic diagram of the gel utilizing solar energy for atmospheric water harvesting and passive evaporative cooling, and vertical cross‐sectional SEM image of the BC/SA/GO‐LiCl hygroscopic aerogel. Reproduced with permission.^[^ ^29^ ^]^ Copyright 2025, Wiley. E,F) Synthesis process of the bidirectionally arranged and layered nanocomposite controlled by vertical temperature gradient (∆T_V_) and radial temperature gradient (∆T_R_), and cross‐sectional SEM images. Reproduced with permission.^[^ ^79^ ^]^ Copyright 2023, Springer Nature.

Conventional freeze‐drying employs natural ice crystal growth to generate porous structures, yet its performance is often constrained by the randomness of pore channels.^[^ ^54^ ^]^ In contrast, directional freeze‐drying utilizes uniaxial cooling to guide ice crystal growth along a specific dimension. Subsequent sublimation results in vertically aligned channel architectures. Benefiting from this “1D channel effect”, the method shortens vapor diffusion paths and significantly enhances adsorption kinetics, particularly under low‐humidity conditions.^[^ ^104^, ^105^ ^]^ For example, Wang et al.^[^ ^106^ ^]^ fabricated SA‐based hygroscopic aerogels via directional freezing. The vertically aligned hierarchical pores served as efficient water transport pathways, reducing diffusion resistance and accelerating sorption/desorption kinetics (**Figure** **11**A,B). Similarly, Cheng et al.^[^ ^29^ ^]^ developed aerogels with precisely aligned vertical channels, achieving a moisture uptake of 1.31 g·g^−1^ at 30% RH within 2 h, with capacity ranging from 1.4 to 5.7 g·g^−1^ across 30–90% RH (Figure 11C,D).

Building upon directional freeze‐drying, biaxial cooling has been introduced to guide ice crystal growth in 2D, resulting in cross‐shaped or grid‐like 3D through‐pore channels.^[^ ^107^, ^108^, ^109^ ^]^ This method enables multi‐objective optimization of mass transfer rate, moisture uptake capacity, and structural stability, making it particularly suitable for extreme humidity conditions. For instance, Wang et al.^[^ ^79^ ^]^ fabricated a hygroscopic aerogel with bidirectionally aligned pore architectures by controlling the orientation of the pre‐freezing temperature field. The well‐ordered hierarchical pore structure significantly reduces diffusion tortuosity and shortens liquid transport distance, synergistically enhancing vapor convection along the vertical direction and radial pore diffusion (Figure 11E,F). Consequently, this hygroscopic aerogel demonstrated an adsorption capacity of 0.9‐6.61 g·g^−1^ under 15%–90% RH, demonstrating both ultrahigh hygroscopic performance and ultrafast kinetics.

Despite these advancements, the fundamental challenge remains the inherent conflict between uncontrolled ice crystal growth and functional requirements. Single‐pore structures fail to balance mesopore‐driven high adsorption capacity with macropore‐driven fast mass transfer, while disordered ice growth causes pore chaos, leading to low‐humidity transport blockage and high‐humidity collapse.^[^ ^110^ ^]^ Addressing these issues requires deeper mechanistic insights into ice crystal regulation and optimized interfacial energy design of materials.

### 4.5 Spinning Technique for HPGs

**Figure 12:**
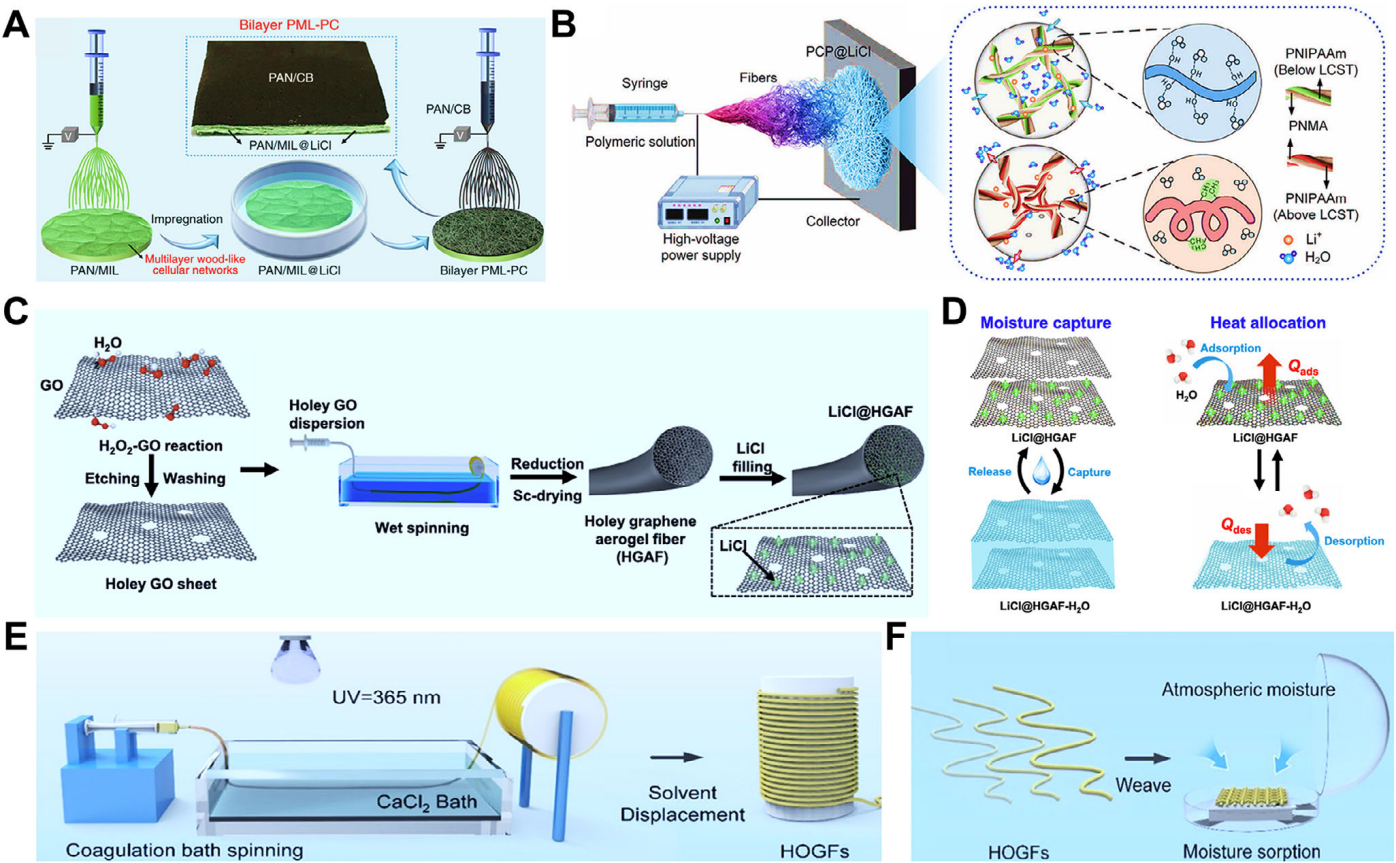
Spinning technique for HPGs. A) Illustration of the spinning technique for HPGs, showing the fabrication process of a biomimetic bilayer PML‐PC nanofiber membrane. PAN/CB nanofibers were electrospun directly onto a multilayer wood‐like cellular substrate composed of PAN/MIL@LiCl nanofibers; the inset displays the resulting bilayer nanofiber membrane. Reproduced with permission.^[^ ^112^ ^]^ Copyright 2020, Springer Nature. B) Fabrication process of the temperature‐responsive PCP@LiCl nanofiber hydrogel. Reproduced with permission.^[^ ^30^ ^]^ Copyright 2024, Wiley. C) Preparation of LiCl@HGAFs aerogel fibers and D) their moisture capture and thermal distribution behavior. The embedded LiCl efficiently absorbs moisture and liquefies it, enabling regeneration through heating for water harvesting. Reproduced with permission.^[^ ^114^ ^]^ Copyright 2024, Springer Nature. E) Schematic of HOGF production via wet‐spinning in a coagulation bath followed by solvent displacement. F) Mechanism of atmospheric moisture adsorption and solar‐driven water release using a HOGFs device. Reproduced with permission.^[^ ^115^ ^]^ Copyright 2023, Springer Nature.

The electrospinning technique allows precise control of fiber diameter (from nanometers to micrometers) and alignment through parameters such as voltage, flow rate, and solvent composition. This enables the fabrication of aligned fiber networks that form continuous and oriented pore channels.^[^ ^111^ ^]^ Such aligned structures enhance water vapor transport via a channel effect, significantly shortening diffusion paths and improving moisture uptake kinetics compared to disordered porous architectures. For example, Ding et al.^[^ ^112^ ^]^ incorporated the metal–organic framework MIL‐101(Cr) into PAN via electrospinning, followed by LiCl crosslinking and a bilayer design to create the PML‐PC nanofiber membrane. These structures achieved a moisture adsorption capacity of 3.01 g·g^−1^ at 90% RH (**Figure** **12**A). Xu et al.^[^ ^113^ ^]^ utilized air‐assisted electrospinning to fabricate multifunctional nanofibers and porous fabrics from SA and PEI, which reduced the RH from 92% to 51.5% within 30 min. In a related approach, Wang et al.^[^ ^30^ ^]^ developed a temperature‐sensitive hydrogel (PCP@LiCl) based on PNIPAAm, combined with carbon nanotubes and LiCl loaded via a self‐crosslinking process. The PNIPAAm component enables hydrophilic–hydrophobic transition near its critical temperature, endowing the material with high moisture uptake across 15–60% RH and rapid water release within 5 min (Figure 12B).

In the field of wet spinning, Zhang et al.^[^ ^114^ ^]^ introduced LiCl into perforated graphene aerogel fibers to fabricate multifunctional gels (LiCl@HGAFs), which achieve a moisture adsorption capacity of 4.14 g·g^−1^ at 90% RH without salt leakage during 6 h (Figure 12C). Chen et al.^[^ ^115^ ^]^ developed hygroscopic organogel fibers (HOGFs) via wet spinning combined with solvent exchange, enabling rapid moisture capture and steam release under light irradiation. These fibers can be woven into 2D atmospheric water harvesting devices with a saturated moisture uptake of 1.63 kg·m^−2^ (Figure 12D). Spinning techniques provide a versatile and robust platform for fabricating high‐performance HPGs with tailored microstructures and multifunctional properties. By facilitating precise control over fiber morphology, pore architecture, and functional integration, these methods play a crucial role in enhancing moisture adsorption and transport kinetics while broadening application adaptability.^[^ ^110^ ^]^ However, scaling these systems to practical applications requires overcoming several challenges: balancing strict process control for structural uniformity, achieving large‐scale manufacturability, and ensuring long‐term stability under repeated hygroscopic cycling and varying operational conditions. Addressing the interplay between precision, scalability, and durability is essential to advance spinning‐based HPGs from laboratory innovation to real‐world implementation.

### 4.6 Phase Separation for HPGs

**Figure 13:**
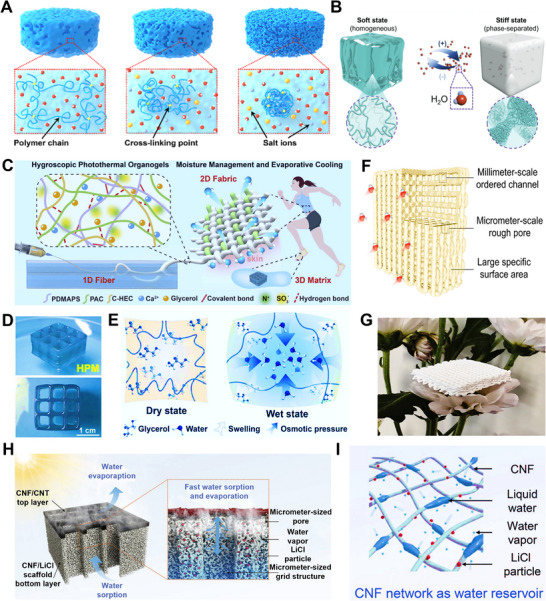
Phase separation and 3D printing for HPGs. A) Salt ion concentrations affect PSBMA chain conformation during photopolymerization, resulting in diverse pore structures. Reproduced with permission.^[^ ^31^ ^]^ Copyright 2025, Wiley. B) Phase separation would be observed from homogenous transparency to phase‐separated opacity. Reproduced with permission.^[^ ^116^ ^]^ Copyright 2022, Wiley. C) Schematic illustration of the preparation of the hygroscopic fibers. D) Digital photographs of 1D fiber and 2D blended fabrics. E) Schematic illustration of water vapor sorption of hygroscopic fibers. Reproduced with permission.^[^ ^117^ ^]^ Copyright 2025, Royal Society of Chemistry. F) Schematics illustrating the 3D structure of CASN‐Li monolith. G) Photograph exhibiting the real morphology of CASN monolith. Reproduced with permission.^[^ ^32^ ^]^ Copyright 2025, Wiley. H) Schematics illustrating the structure and working principle of the 3D printed bilayer scaffold sorbents. I) Schematic illustrating CNF scaffold acting as a water reservoir to store the absorbed water. Reproduced with permission.^[^ ^118^ ^]^ Copyright 2024, Wiley.

Phase separation plays a key role in determining the microstructure and properties of gels. Fundamentally, it involves disrupting the thermodynamic compatibility between polymer and solvent through kinetic or thermodynamic interventions, driving the system to spontaneously separate into polymer‐rich and solvent‐rich phases.^[^ ^110^, ^119^ ^]^ When in contact with water molecules, the chain segments swell and the originally hidden hydrophilic groups are exposed on the pore surface as the chain segments stretch, forming “accessible hydrophilic sites” and thus increasing the exposure of hydrophilic sites. Precise control over this process allows not only for increased specific surface area—providing more active sites for water adsorption—but also for optimization of moisture diffusion pathways within the gel. This leads to synergistic enhancements in moisture uptake, structural stability, and functional diversity (**Figure** **13**A).^[^ ^31^ ^]^ For example, Zhu et al.^[^ ^116^ ^]^ introduced ionic liquids and lithium salts into the poly (benzyl methacrylate) (PBzMA) gel network (Figure 13B). Upon contact with moisture, it can activate polymer intermolecular interactions and undergoes phase separation. Under 10–90% RH, the gel demonstrated remarkable stability in storage and loss moduli, alongside a humidity‐triggered shape memory effect. Temperature also serves as a critical regulator of phase separation. For example, PNIPAM exhibits hydrophilic swelling below its LCST and hydrophobic contraction above it, enabling dynamic microstructural control in response to thermal stimuli.^[^ ^27^, ^93^, ^94^ ^]^ Despite the advantages for designing high‐performance HPGs, phase separation is highly sensitive to thermodynamic and kinetic conditions, often resulting in structural heterogeneity.^[^ ^120^ ^]^

### 4.7 3D Printing for HPGs

3D printing technology enables precise regulation of pore size, distribution, and connectivity via computer‐aided design, facilitating the construction of gels with gradient pore structures (e.g., surface micropores and inner macropores) or bionic hierarchical architectures (resembling sponge or honeycomb topologies).^[^ ^53^ ^]^ This capability significantly optimizes vapor diffusion pathways and enhances moisture adsorption efficiency. For instance, Xu et al.^[^ ^117^ ^]^ fabricated HPGs with multi‐scale porous structures via 3D printing, improving moisture uptake performance while minimizing material usage (Figure 13C–E). By employing a multi‐network crosslinking strategy with two hygroscopic polymers, the team further developed multidimensional hygroscopic materials applicable to efficient humidity management and evaporative cooling. Wang et al.^[^ ^32^ ^]^ developed a bionic porous HPGs (CASN‐Li) via direct ink writing 3D printing technology (Figure 13F). Its macroscopically ordered mass transfer channels and micron‐scale pores synergistically accelerate water transport, with a moisture adsorption rate reaching 1.5 g·g^−1^ at 90% RH. CASN‐Li leverages low‐cost components like SA and hydrophilic fumed silica, realizing structural customization and batch processing through 3D printing with controllable large‐scale production costs (Figure 13G). Jiang et al.^[^ ^118^ ^]^ constructed a dual‐layer porous absorber composed of nanocellulose/lithium chloride and nanocellulose/carbon nanotubes by integrating 3D printing with freeze‐drying (Figure 13H). This hierarchical pore design offers a novel approach to prevent salt leakage while promoting accelerated vapor adsorption and release. This methodology overcomes the limitations of disordered pore structures typical of conventional freeze‐drying, upgrading the traditional ice‐templating mechanism from passive pore formation to actively designed architectures leveraging the digital programmability of 3D printing (Figure 13I). Although 3D printing offers an innovative pathway for structural customization and functional integration of HPGs, its broad adoption remains constrained by challenges such as material compatibility, production efficiency, and cost‐effectiveness.

In sum, the preparation methods of HPGs exhibit significant diversity, encompassing techniques such as physical foaming, freeze‐drying, 3D printing, sol‐gel, polymer crosslinking, spinning, and phase separation.^[^ ^110^ ^]^ The core advantages and disadvantages of each method in terms of pore structure control precision, scalability, mechanical properties, and suitable application scenarios have been systematically summarized in **Table** **1**. For example, the physical foaming method, with its simple process, low cost, and high pore connectivity, has strong potential for large‐scale production, but its ability to control pore orientation is weak, making it more suitable for applications such as building dehumidification and industrial pipeline humidity control, where the directionality of pores is less critical.^[^ ^100^ ^]^ The freeze‐drying method can precisely construct vertically or horizontally oriented channels through temperature gradients, performing excellently in low‐humidity atmospheric water collection scenarios, but it is limited by batch production and high energy consumption, making it difficult to meet low‐cost large‐scale demands.^[^ ^54^ ^]^ 3D printing can achieve customized structures such as gradient channels and lattice channels, perfectly matching the mass transfer requirements in scenarios like precision electronic electrolytes, yet it suffers from high equipment costs.^[^ ^118^ ^]^ Therefore, in practical applications, it is necessary to comprehensively weigh the core requirements of the target scenario—such as mass transfer efficiency, structural customizability, and mechanical stability—to achieve a precise match between the hygroscopic gel performance and application requirements.

**Table 1:** The construction strategy of different HPGs.

Construction strategy	Pore adjustment capability	Applicable	Disadvantages
**Sol–Gel**	The pore size, pore morphology, and surface functional groups can be regulated at the molecular level to optimize moisture absorption sites and diffusion channels.^[^ ^96^ ^]^	Scenarios requiring precise regulation of pore characteristics to enhance moisture absorption efficiency, such as rapid moisture capture in high‐humidity environments.^[^ ^26^, ^96^ ^]^	The reaction conditions (e.g., pH, temperature) require strict control, leading to a complex process.
**Polymerization crosslinking**	Complex structures such as interpenetrating polymer networks (IPNs) can be constructed by doping functional components to regulate the density and connectivity of pore networks.^[^ ^27^, ^59^ ^]^	The moisture absorption, water retention, and application of spontaneous water release or photothermal responsive water release require the synergy of pore networks and hydrophilic groups.^[^ ^45^, ^97^ ^]^	Unreacted monomers or initiators may remain, affecting biocompatibility and stability.^[^ ^11^, ^99^ ^]^
**Physical foaming**	A hollow spherical porous structure with high connectivity can be constructed. The pore size is mostly in the micrometer range.^[^ ^28^, ^83^ ^]^	Atmospheric water harvesting in low‐to‐medium humidity environments and scenarios requiring rapid adsorption‐desorption kinetics.^[^ ^100^ ^]^	There is a trade‐off between high porosity and mechanical strength, which limits its application in load‐bearing scenarios.
**Freeze–drying**	Ordered pore channels can be prepared by controlling the ice crystal growth direction (unidirectional/ bidirectional freezing).^[^ ^79^, ^109^ ^]^	High‐efficiency moisture absorption in low‐humidity environments and scenarios requiring rapid mass transfer.^[^ ^55^, ^110^ ^]^	Regular freezing tends to form disordered pores, while moisture absorption rate decreases under low humidity; repeated cycles under high humidity may easily lead to pore collapse.^[^ ^54^, ^104^ ^]^
**Spinning technique**	Nano‐ to micro‐scale fibers can be fabricated, which form continuous moisture transport channels through directional alignment.^[^ ^30^, ^112^ ^]^	Flexible devices and scenarios requiring high specific surface area and short diffusion paths.^[^ ^111^, ^115^ ^]^	Electrospinning has low productivity, while wet spinning requires large amounts of coagulation bath. Fibers tend to agglomerate, clogging pores and reducing diffusion efficiency.^[^ ^110^ ^]^
**Phase separation**	Spontaneous phase separation between the polymer and solvent can be achieved by adjusting temperature or solvent. The pore size is mostly in the micrometer range.^[^ ^31^, ^94^ ^]^	Scenarios requiring humidity‐responsive shape memory or mechanical property regulation, and applications needing heterogeneous structure construction to enhance moisture absorption selectivity.^[^ ^120^ ^]^	The phase separation process is susceptible to kinetic and thermodynamic factors, resulting in uneven pore distribution and significant performance fluctuations.^[^ ^27^, ^93^ ^]^
**3D printing**	The pore size (ranging from micrometers to millimeters), distribution, and connectivity can be precisely regulated, enabling the fabrication of gradient porous structures or bionic structures.^[^ ^32^, ^121^ ^]^	For the customized design of moisture‐absorbing devices and their adaptive applications in complex scenarios.^[^ ^53^ ^]^	This method limits the application of high‐viscosity polymers; high‐precision printing is slow, while high‐speed printing tends to cause poor interlayer bonding.^[^ ^53^, ^118^ ^]^

## 5 Recent Advances in the Application of HPGs

### 5.1 Application in AWH

**Figure 14:**
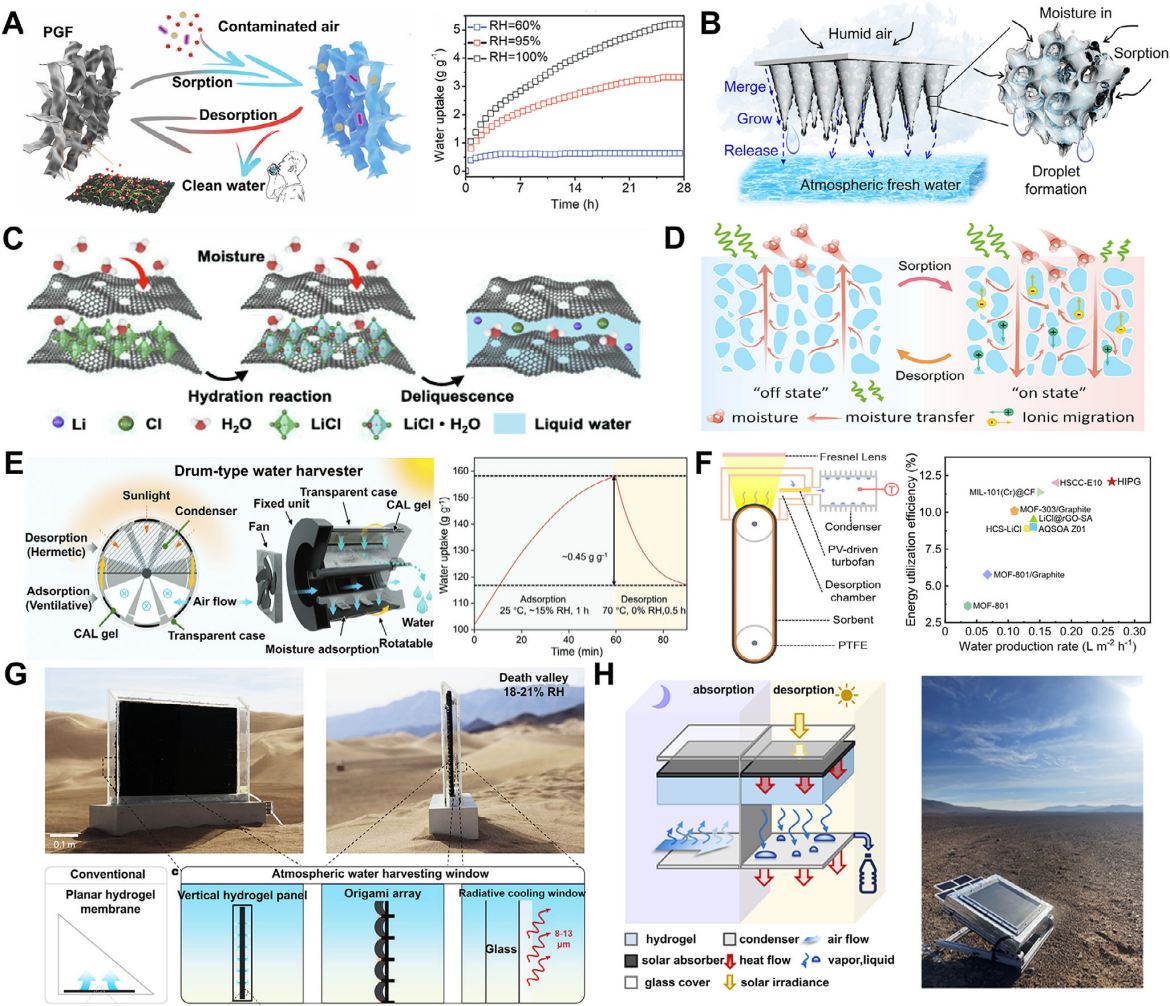
Application in AWH. A) Moisture harvesting from contaminated air and solar‐driven desorption for clean water production using the PGF. Insets: interaction mechanism between water molecules and the PGF (left), and kinetic uptake profiles at 60%, 95%, and 100% RH (right). Reproduced with permission.^[^ ^101^ ^]^ Copyright 2020, Wiley. B) Structural schematic and optical image of the PCA‐MOF cone array. Reproduced with permission.^[^ ^98^ ^]^ Copyright 2020, American Association for the Advancement of Science. C) Mechanism of moisture adsorption process. Reproduced with permission.^[^ ^114^ ^]^ Copyright 2022, Springer Nature. D) Reversible switching between "off‐state" and "on‐state" of hygroscopic LiCl@GA. Reproduced with permission.^[^ ^123^ ^]^ Copyright 2025, Wiley. E) Solar‐driven drum‐type harvester enabling continuous multi‐cycle operation (left), and adsorption/desorption performance of CAL gel at 15% RH. Reproduced with permission.^[^ ^94^ ^]^ Copyright 2024, Wiley. F) Schematic of the continuous solar‐assisted AWH device (left) and comparative water production performance. Reproduced with permission.^[^ ^83^ ^]^ Copyright 2024, Springer Nature. G) Architecture of the AWH device and field water collection in Death Valley. Reproduced with permission.^[^ ^124^ ^]^ Copyright 2025, Springer Nature. H) System diagram with key components and energy/mass flow during day–night operation (left), and deployment view of the AWH system in the Atacama Desert, Chile (right). Reproduced with permission.^[^ ^125^ ^]^ Copyright 2025, Cell Press.

Atmospheric Water Harvesting (AWH) is a cutting‐edge technology for extracting freshwater from ubiquitous atmospheric moisture. It exhibits broad application prospects and offers a practical technical pathway to alleviate global water scarcity. Compared with conventional water sources, atmospheric moisture has the distinct advantage of being geographically unconstrained—this not only enables decentralized deployment of AWH, but also makes it a key supplement to existing water supply systems. Among materials for AWH, HPGs have become a highly attractive core material platform, owing to their highly tunable microstructures, unique swelling response, and excellent functional integration capacity.^[^ ^122^ ^]^ For instance, Qu et al.^[^ ^101^ ^]^ everaged the strong electrostatic interactions between ─COOH groups and water molecules, enabling the gel to achieve a hygroscopic capacity of 0.64–5.2 g·g^−1^ at 60–100% RH (**Figure** **14**A). Ho et al.^[^ ^98^ ^]^ introduced metal coordination sites into the gel framework, facilitating efficient water capture with a moisture uptake of 6.0 g·g^−1^ at 90% RH (Figure 14B). To enhance performance under low‐humidity conditions, polyelectrolytes can be incorporated into the polymer network. The resulting large osmotic pressure gradient promotes rapid inward transport of adsorbed water molecules, freeing up surface sites for continued adsorption and thereby improving overall hygroscopic kinetics. For instance, the polyelectrolyte hydrogel developed by Tan et al.^[^ ^71^ ^]^ exhibited high internal osmotic pressure and coordinated polymer–salt interactions. This design enables continuous moisture migration into the gel interior, yielding a hygroscopic capacity of 0.65–1.00 g·g^−1^ at 15–90% RH. ​

Beyond polyelectrolyte introduction, constructing porous gel systems or reducing gel dimensionality is another effective approach to enhance low‐humidity hygroscopic performance. Zhang et al.^[^ ^114^ ^]^ performed etching treatment on hygroscopic graphene aerogel fibers, which significantly enhanced their hygroscopic capacity, with a moisture absorption amount of 0.66 g·g^−1^ at 30% RH(Figure 14C). Huang et al.^[^ ^123^ ^]^ developed a lithium chloride@graphene aerogel (LiCl@GA). Benefiting from the ordered pore structure formed by temperature gradient‐induced ice crystal growth, LiCl@GA has a moisture absorption capacity of 0.69 g·g^−1^ at 30% RH (Figure 14D). Furthermore, modulating the interactions between polymer chains and water molecules has proven effective in improving moisture transport. For instance, Chen et al.^[^ ^94^ ^]^ incorporated thermoresponsive hydroxypropyl cellulose into the gel system. By exploiting polymer conformational changes to regulate water phase behavior, the gel achieved a hygroscopic capacity of 0.45 g·g^−1^ at 15% RH (Figure 14E).

Regarding desorption performance, photothermal conversion materials are critical for enhancing desorption efficiency.^[^ ^126^ ^]^ Among inorganic photothermal materials, advanced options such as plasmonic nanoparticles^[^ ^127^ ^]^ and CNTs^[^ ^128^, ^129^ ^]^ have been integrated into HPGs to achieve efficient solar‐to‐thermal conversion. For organic photothermal modification, constructing a conjugated PPy‐Cl polymer interpenetrating network within the gel can endow HPGs with excellent photothermal property.^[^ ^27^ ^]^ These photothermal components absorb solar energy and convert it into thermal energy, which not only provides localized heat sources for desorption but also reduces overall energy consumption. Additionally, the introduction of alternating hydrophilic‐hydrophobic microdomains into HPGs leverages interfacial tension gradients—this drives the rapid detachment of adsorbed water from the gel surface, thereby promoting the migration and release of adsorbed water and further enhancing cycling efficiency.^[^ ^130^ ^]^ While the above strategies effectively enhance the desorption and cycling performance of HPGs, it should be emphasized that each type of HPGs has its specific optimal application scenario. Therefore, selecting appropriate HPGs based on different application scenarios is more practical and reliable than developing HPGs with broad applicability and superior performance.

From a device design perspective, integrating optimized materials with rational device structures is key to enhancing overall AWH efficiency, particularly condensation performance. For general outdoor scenarios, Wang et al.^[^ ^83^ ^]^ developed a continuous solar‐driven AWH device powered by light and wind. Equipped with separated desorption and condensation chambers, the device not only minimizes light loss caused by water droplets but also prevents radiant heating of the condensing surface—these improvements collectively reduce the condensation temperature and boost the water collection rate. Moreover, the device utilizes natural wind to drive the cyclic rotation of the adsorption bed, thereby enabling fully renewable operation. Under the experimental conditions, the ambient temperature was ≈27–29 °C, the environmental RH fluctuated between 25.6% and 33.5%, and the solar irradiation intensity received by the sorbent (concentrated via a Fresnel lens) was ≈3.5–4 suns. This device achieved a daily water production rate of 3.5–8.9 L·m^−2^·day^−1^ (Figure 14F). For extreme climates(e.g., high‐temperature arid areas), Zhao et al.^[^ ^124^ ^]^ encapsulated a highly hygroscopic hydrogel within a glass cavity coated with a radiative cooling layer. The hydrogel surface features closely packed dome‐shaped structures—similar to bubble wrap—that significantly increase the air‐contact area, thus improving moisture adsorption efficiency even in high‐temperature, low‐humidity conditions. This device demonstrated stable operation in harsh environments such as Death Valley, producing 57.0‐161.5 mL·device^−1^·day^−1^ (Figure 14G). For ultra‐arid regions (where humidity is extremely low), Fil et al.^[^ ^125^ ^]^ developed a solar‐driven hydrogel device using a PAM‐LiCl composite as the core sorbent. By optimizing structural parameters, the system attained a thermal efficiency of 16% and a maximum daily water yield of 1.7 L·m^−2^·day^−1^, enabling effective freshwater production even in the Atacama Desert. The test cycle of this device consists of 12 h of water absorption and 8 h of water desorption. For the freshwater produced, the concentrations of silver, magnesium, indium, iron, copper, and potassium were all significantly below the acceptable levels for human consumption. However, in the water samples collected from outdoor experimental tests, lithium and aluminum were present at concentrations slightly exceeding the acceptable limits for drinking water (Figure 14H). Collectively, these HPGs‐based AWH devices address the challenges of different arid environments by targeted structural designs; they not only exhibit high water production rates and cyclic stability but also produce liquid water that meets World Health Organization drinking standards. Moving forward, by further optimizing sorption‐desorption kinetics and scaling up device architecture, HPGs show great promise for practical AWH applications in arid regions.

HPGs exhibit considerable potential for AWH, with key strengths including robust moisture uptake across varying humidity levels, efficient low‐energy desorption (e.g., via photothermal conversion), and enhanced adaptability to extreme environments. This adaptability is achieved through structural modifications (e.g., incorporation of carboxyl groups, metal coordination sites, and porous frameworks), compositional tuning (e.g., addition of polyelectrolytes, hygroscopic salts, and photothermal agents), and device‐level innovations (e.g., segregated reactor designs and radiative cooling coatings). Despite these notable merits that underpin their viability for AWH, their transition from laboratory research to large‐scale practical application faces multi‐faceted challenges.^[^ ^131^, ^132^, ^133^ ^]^ From a material‐performance perspective, low‐humidity conditions pose a fundamental trade‐off between moisture absorption kinetics and capacity: porous structures with high surface area are prone to pore collapse, while the incorporation of hygroscopic salts—though enhancing moisture absorption capacity—often results in salt crystallization, leakage, and potential water contamination; both issues further compromise the reliability and safety of HPGs‐based systems in large‐scale operations. In terms of desorption efficiency, uneven distribution of photothermal materials can lead to localized overheating and reduced energy efficiency. Additionally, solar‐dependent desorption is inherently intermittent under low‐light conditions, often necessitating auxiliary energy sources that incur additional operational costs. For device scaling‐up, additional difficulties emerge, including non‐uniform mass and heat transfer, high costs of advanced materials, and complex manufacturing processes—all of which contribute to elevated unit water production costs.^[^ ^28^, ^90^, ^134^ ^]^ Regarding environmental adaptability and long‐term reliability, moisture absorption capacity remains inadequate under extreme environmental conditions. Long‐term cycling further presents challenges such as delamination at the gel‐device substrate or gel‐photothermal material interfaces, as well as mechanical degradation of device components (e.g., rotating parts and radiative cooling coatings) in harsh environments.^[^ ^33^, ^135^, ^136^ ^]^ Targeted breakthroughs in these interrelated areas are essential to advance the practical deployment of HPGs‐based AWH systems.

### 5.2 Application in Electricity Production

**Figure 15:**
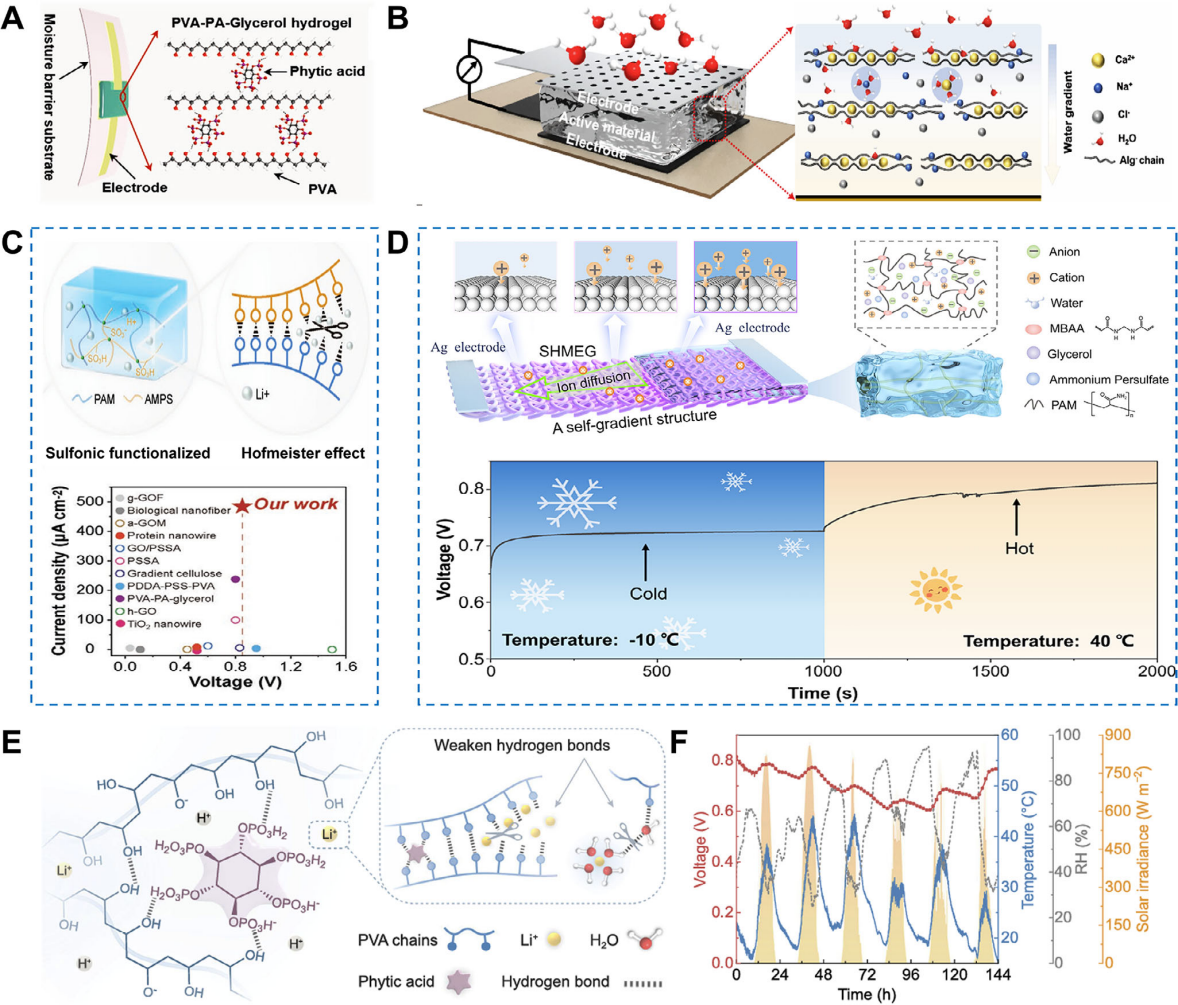
Application in Electricity Production. A) Schematic and electrical output characteristics of the IHMEG. Reproduced with permission.^[^ ^138^ ^]^ Copyright 2022, Wiley. B) Structure of a single MEG unit. The active material consists of a supramolecular Alg‐Ca/Na network within a PVA hydrogel, formed through partial substitution of Na^+^ with Ca^2+^ ions acting as crosslinking points in an egg‐box structure. The moisture gradient is defined as the difference in water content between the upper and lower surfaces of the MEG. Reproduced with permission.^[^ ^143^ ^]^ Copyright 2024, Springer Nature. C) Structural details of the MEG along with a molecular engineering strategy and corresponding output performance. Reproduced with permission.^[^ ^139^ ^]^ Copyright 2023, Wiley. D) Preparation, structure, and low‐temperature performance at 75% RH of the SHMEG. Reproduced with permission.^[^ ^142^ ^]^ Copyright 2025, Wiley. E) Material composition and intermolecular interactions within the ionic hydrogel. F) Continuous open‐circuit voltage output of the PP/IH device recorded over six days in an outdoor environment. Reproduced with permission.^[^ ^144^ ^]^ Copyright 2024, Springer Nature.

Moisture‐enabled power generation represents an emerging technological frontier in renewable energy, distinguished by the ability to harness the ubiquitous chemical potential of atmospheric water. Among various material platforms, HPGs have emerged as ideal candidates for this purpose. Their operation relies on the continuous process of atmospheric moisture adsorption, which is followed by ion dissociation and migration within the gel network, culminating in the efficient conversion of hygroscopic energy into electrical output.^[^ ^137^ ^]^ ​Substantial efforts have been devoted to enhancing power output through innovative material design. For example, Tao et al.^[^ ^138^ ^]^ developed a PVA‐based ionic hydrogel (IHMEG) that achieves a power density of 35 µW cm^−2^, leveraging its high moisture uptake and rapid ion transport (**Figure** **15**A). To further increase current density, the same team designed an HPGs‐based moisture generator capable of spontaneously absorbing vapor to form an asymmetric humidity gradient. This promoted ion dissociation and diffusion from the polymer network, resulting in electrical energy output with a power density as high as 0.11 mW cm^−^^2^ (Figure 15B). Similarly, Tang^[^ ^139^ ^]^ utilized lithium ions to induce a Hofmeister effect among polymer chains, reducing energy barriers for proton transport, effectively constructing a proton “highway”, and endowing the hydrogel with exceptional proton conductivity. The resulting generator achieved a short‐circuit current density of 480 µA cm^−2^.​

To address the critical issue of power output degradation in HPGs after moisture saturation, Tan et al.^[^ ^140^ ^]^ developed a PVA hydrogel featuring an asymmetric moisture sorption structure. Under specific humidity conditions, the surface of this PVA hydrogel spontaneously forms a stable “wet‐dry” gradient. Due to the confinement effect of the polymer network in the hydrated region, adsorbed water molecules are prevented from diffusing freely toward dry zones. As a result, the hydrogel maintains a stable open‐circuit voltage for hundreds of hours even after reaching moisture saturation. Operational stability under extreme conditions constitutes another critical requirement for practical application. Li et al.^[^ ^141^ ^]^ leveraged preferential hydrogen bonding between glycerol and water to disrupt native water‐water interactions and inhibit ice crystallization. The resulting low‐temperature hydrogel retains ion dissociation and mobility even at −35 °C (Figure 15C). Similarly, Qin et al.^[^ ^142^ ^]^ constructed an electric double‐layer‐based hygroscopic generator (SHMEG) by diffusing an ionic polyacrylamide hydrogel onto carbon‐fiber knit fabric, forming an intrinsic moisture gradient. Combined with the anti‐freezing and anti‐drying properties of a glycerol–water binary solvent, the device achieves stable power generation across a broad range of conditions (−10–40  °C, 10–90% RH, Figure 15D). At room temperature (≈25 °C) and ≈75% RH, the SHMEG exhibits a sustained voltage output of 0.75 V for 140 h and a current output of 15 µA. Owing to its high hygroscopicity, moisture retention capacity, and temperature adaptability, the SHMEG can reliably provide stable electrical output, with 0.5 V at 20% RH and 0.7 V at −10 °C.

To enable stable and continuous cycling in moisture‐driven energy systems, recent efforts have focused on integrating adsorption and desorption processes to sustain water and ion transport beyond single moisture capture events.^[^ ^145^, ^146^ ^]^ In addition to traditional humidity gradients, natural energy sources such as solar radiation and ambient temperature variations have been strategically employed to promote cyclic operation, offering a sustainable pathway for long‐term power generation. For example, Lai et al.^[^ ^147^ ^]^ developed a light‐enhanced moisture‐induced power generator using a PAAm‐phytic acid hydrogel incorporated with the photosensitizer Erythrosin B. his system delivers enhanced electrical output under synergistic photo‐moisture conditions, enabling all‐weather operation regardless of solar intensity. Zhao et al.^[^ ^144^ ^]^ developed a bilayered polymer system (PP/IH) consisting of a hydrophobic porous top layer and a bottom ionic hydrogel layer with high hygroscopicity. The hydrogel spontaneously captures moisture and facilitates ion dissociation, while water vapor is released through the upper porous membrane. This continuous adsorption–evaporation process sustains a steady ion flux through negatively charged nanochannels (Figure 15E). Furthermore, the hierarchically porous top layer contributes to radiative cooling, which reduces solar heat absorption—thereby suppressing excessive daytime evaporation that could diminish humidity gradients—while simultaneously enhancing nighttime moisture uptake by lowering the material's temperature (creating a temperature difference with the ambient air that drives moisture adsorption). This rationally integrated structure enables stable day–night cycling, achieving a power density of 51 µW cm^−^^2^ under 70% RH and maintaining six days of outdoor operation (Figure 15F).

Despite significant progress in moisture‐enabled power generation using HPGs—which have led to breakthroughs in power density, short‐circuit current density, and cycling continuity, their practical application still faces several critical challenges.^[^ ^137^, ^140^ ^]^ On the one hand, the power output of most current HPGs‐based generators remains on the order of microwatts to mill watts, which is insufficient to meet the energy demands of the majority of practical electronic devices. Enhancing energy conversion efficiency within limited volumes and areas remains a primary obstacle to commercialization. Moreover, repeated adsorption–desorption cycles often result in gel network fatigue, ion loss, phase separation, and interfacial delamination, significantly compromising the device's service life and operational reliability. Furthermore, desorption modes reliant on natural energy sources such as solar power are highly susceptible to weather conditions, resulting in intermittent power generation and poor stability.^[^ ^148^, ^149^ ^]^ Achieving autonomous, weather‐independent, stable, and continuous energy output across diverse environmental conditions still requires system‐level innovation and interdisciplinary solutions.

### 5.3 Application in Thermal Management

**Figure 16:**
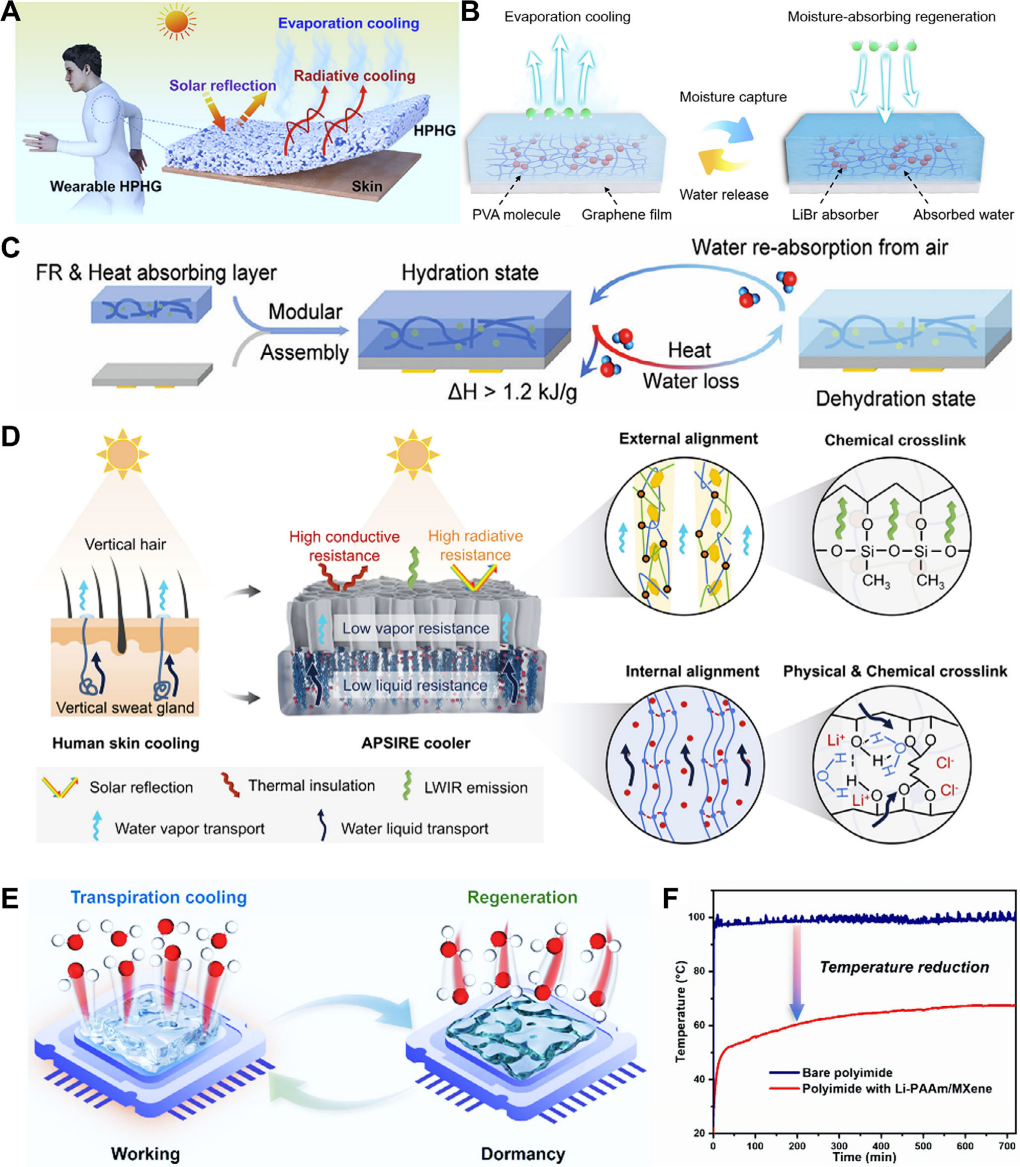
Application in Thermal Management. A) Schematic illustration of the HPHG applied for wearable passive radiative and evaporative cooling under extreme heat. Reproduced with permission.^[^ ^152^ ^]^ Copyright 2024, Cell Press. B) Working mechanism of the SPG cooling film. Reproduced with permission.^[^ ^153^ ^]^ Copyright 2025, Wiley. C) High‐enthalpy thermal dissipation through hydrogel encapsulation: whereas conventional elastomer substrates accumulate heat and risk combustion, this hydrogel‐based layer provides cooling through moisture evaporation and regenerates via vapor re‐adsorption. Reproduced with permission.^[^ ^154^ ^]^ Copyright 2024, Wiley. D) Bioinspired multiscale design of the ASPIRE cooler mimicking the structure of human skin with vertically aligned sweat glands and hairs. Reproduced with permission.^[^ ^155^ ^]^ Copyright 2025, Springer Nature. E) Transpiration cooling and regeneration mechanism of the LiBr‐PAAm/MXene hydrogel. F) Temperature comparison of a simulated heat source with and without transpiration cooling under 1500 W·m^−2^ heat flux. Reproduced with permission.^[^ ^19^ ^]^ Copyright 2025, Wiley.

The increasing frequency of extreme high‐temperature events under global climate change has intensified the demand for personal thermal management technologies, which leverages their reversible moisture sorption‐desorption cycles, high latent heat of vaporization, and highly tunable structural and functional properties.^[^ ^150^, ^151^ ^]^ For example, Chen et al.^[^ ^115^ ^]^ developed hygroscopic organic gel fibers with a saturated water absorption capacity of 1.63 kg·m^−2^ and a solar‐driven moisture release rate of 1.46 kg·m^−2^·h^−1^. These fibers function not only as self‐sustaining freshwater harvesters but also as wearable dehumidifiers, reducing skin microenvironment humidity from 90% to 33.4% within 12.5 min. Beyond moisture regulation, certain HPGs incorporate high‐emissivity components to radiate heat directly into outer space (≈3 K). A notable example is the work by Wang et al.^[^ ^152^ ^]^ who synthesized a lightweight hybrid hydrogel (HPHG) by embedding superhydrophobic silica aerogels within a 3D hydrogel network via cyclic freeze‐thaw processing. The Si‐O‐Si vibrational bands in HPHG exhibit strong thermal emission within the 7–14 µm band—closely aligned with the atmospheric window (8–13 µm)—while maintaining low visible light absorption. A hierarchical pore structure further enhances solar reflectance, effectively minimizing solar heat gain. This material achieves a surface temperature 22.5  °C below ambient and sustains cooling for up to 15 h, outperforming conventional radiative coolers (**Figure** **16**A).

In electronics cooling, HPGs dissipate waste heat through the evaporation of absorbed moisture. Liu et al.^[^ ^35^ ^]^ demonstrated a polyacrylamide hydrogel that boosted polycrystalline solar cell efficiency by 1.5% through rapid evaporative cooling and self‐regeneration. Similarly, Chen et al.^[^ ^72^ ^]^ introduced the ionic liquid 1‐ethyl‐3‐methylimidazolium acetate ([EMIM]^+^[Ac]^−^) into a gel network to engineer a high‐performance HPG. This HPG not only provides significantly improved and prolonged evaporative cooling for devices but also regenerates its cooling performance via atmospheric moisture absorption. To enhance functionality through structural design, Chen et al.^[^ ^154^ ^]^ employed a hydrogel encapsulation strategy, where water evaporation forms a protective barrier capable of preventing flame penetration for over 10 s. This strategy not only enhances flame retardancy but also maintains effective heat dissipation through evaporation, supporting stable operation of stretchable electronics under extreme conditions. Bi et al.^[^ ^153^ ^]^ developed a self‐hygroscopic polyvinyl alcohol/graphene (SPG) cooling film with excellent performance. In outdoor testing, the SPG film reduced the temperature of a photovoltaic cell by 20.6  °C—lowering thermal losses that degrade efficiency—and increased its average output power from 74 to 93 W m^−^^2^ – an improvement of ≈25.7% (Figure 16B). Beyond passive cooling, HPGs are integrated with responsive functions for process monitoring. Chen et al.^[^ ^156^ ^]^ designed a self‐hygroscopic smart color‐changing HPG. By modulating the water molecular microenvironment through interactions between polymer chains and functional ions—weakening the binding forces around water molecules and lowering the gel's evaporation barrier—this hydrogel enables rapid device cooling. Simultaneously, leveraging the color changes of cobalt ions, it provides intuitive visual feedback on the evaporative cooling potential and real‐time cooling status of the heat sink, offering dynamic visual monitoring of the dissipation process (Figure 16C).

To integrate multiple cooling mechanisms for higher efficiency, Shen et al.^[^ ^155^ ^]^ designed a dual‐layer cooling system: a moisture‐regenerating inner hydrogel facilitates daytime evaporation and nighttime rehydration, while an outer aerogel (composed of vertically aligned hydrophobic boron nitride nanosheets in a carboxymethyl cellulose (CMC)‐PVA matrix) enhances radiative dissipation, reduces solar absorption, and minimizes vapor diffusion resistance. This integrated mechanism combines insulation, radiation, and evaporation for high‐efficiency cooling (Figure 16D). Wen et al.^[^ ^19^ ^]^ fabricated a lithium bromide‐polyacrylamide/MXene (LiBr‐PAAm/MXene) hydrogel (MXene refers to a class of 2D transition metal carbides/nitrides) in which MXene nanosheets form hydrogen‐bonded thermal bridges within the PAAm network, thereby significantly improving thermal conductivity. The addition of LiBr enhances moisture uptake, enabling autonomous water harvesting and cyclic transpiration cooling (Figure 16E,F). In a complementary study, Ji et al.^[^ ^157^ ^]^ developed a humidity–color dual‐responsive PADAL (Polyacrylamide‐Dopamine‐Aluminum) hydrogel that visually indicates its hydration state through intrinsic color shifts, offering both high hygroscopic performance and straightforward real‐time monitoring of absorption–desorption dynamics.

Despite these remarkable advances, challenges in mechanical durability and long‐term stability remain critical barriers to practical deployment. The high water content required for cooling often compromises mechanical strength, leading to network damage and functional failure under repeated stress. Furthermore, hygroscopic components are prone to degradation via oxidation or leaching, and some matrix structures deteriorate over multiple sorption‐desorption cycles.^[^ ^160^, ^161^ ^]^ These issues ultimately impair the cycling sustainability and reliability of thermal management systems. Therefore, future research must focus on developing mechanically robust, environmentally stable HPGs through innovative network designs—such as double‐network hydrogels, nanocomposite reinforcement, and the integration of self‐healing mechanisms—to ensure performance longevity and operational reliability under real‐world conditions.

### 5.4 Application in Fuel Production

Hydrogen is a clean and versatile energy carrier, and solar‐driven overall water splitting is an ideal pathway for its sustainable production.^[^ ^160^ ^]^ However, conventional photocatalytic systems are geographically constrained by their dependence on liquid water. HPGs offer a transformative solution by directly harnessing ubiquitous atmospheric humidity as a water source. This capability significantly expands the geographical applicability and resource availability of hydrogen production technologies. Furthermore, their 3D porous network efficiently captures and concentrates water molecules from air, while simultaneously acting as a host for electrolytes. This creates a hydrated, ion‐conductive microenvironment that enables efficient water electrolysis, ensuring sustained hydrogen production from ambient air.^[^ ^161^ ^]^

**Figure 17:**
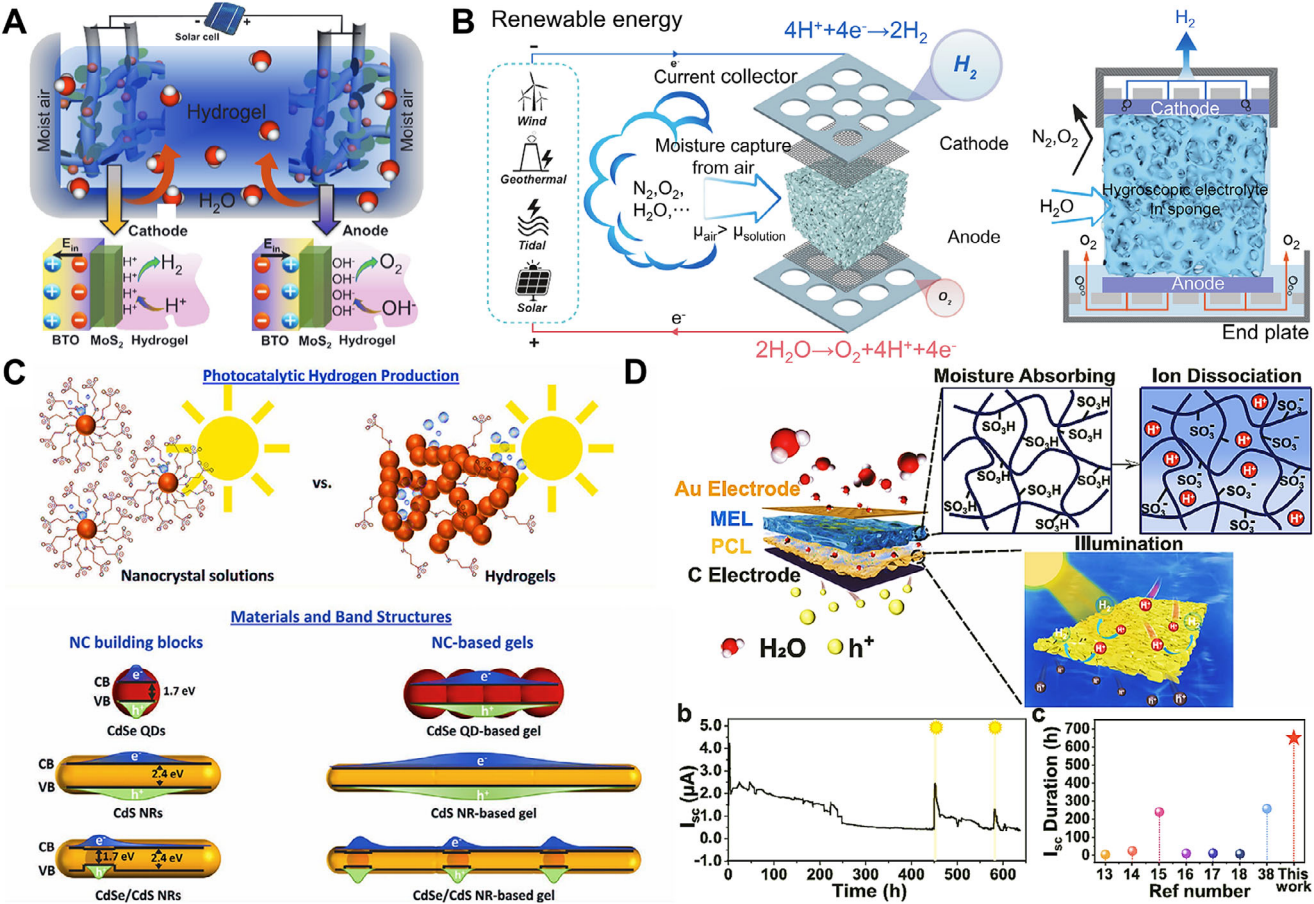
Application in Fuel Production. A) Mechanism of humidity splitting with a cathode of negatively polarized BTO@MoS_2_ and an anode of positively polarized BTO@MoS_2_. Reproduced with permission.^[^ ^162^ ^]^ Copyright 2020, Wiley. B) Structure of the DAE module incorporating a water harvesting unit composed of a porous medium infused with hygroscopic ionic solution. Reproduced with permission.^[^ ^161^ ^]^ Copyright 2022, Springer Nature. C) Photocatalytic hydrogen production system illustrating material composition and band structures of nanocrystal (NC) building blocks and NC‐based gels. Phase transfer from organic to aqueous phase is followed by ligand oxidation with H_2_O_2_, enabling photocatalytic H_2_ generation in both aqueous dispersions and hydrogels. Reproduced with permission.^[^ ^166^ ^]^ Copyright 2023, Wiley. D) Design and performance overview of the MEG device. Reproduced with permission.^[^ ^167^ ^]^ Copyright 2025, Springer Nature.

To validate this potential, several research teams have developed HPGs for efficient hydrogen production. In a previous study, Tan et al.^[^ ^162^ ^]^ developed an efficient electrocatalytic water‐splitting system by integrating ferroelectric barium titanate@molybdenum disulfide (BaTiO_3_@MoS_2_) hybrid catalysts into the hydrogel. Within this architecture, the BaTiO_3_@MoS_2_ hydrogel continuously captures atmospheric moisture, serving as a self‐sustaining water reservoir, while the polarization of BaTiO_3_@MoS_2_ is effectively activated, markedly facilitating interfacial charge transfer.This synergistic mechanism enables the hybrid catalyst to exhibit remarkable electrocatalytic performance in both the hydrogen evolution reaction and peroxide formation. Building on this, the same team further introduced cuprous oxide@barium titanate (Cu_2_O@BaTiO_3_) photocatalysts into HPGs. Beyond acting as“moisture‐absorbing hands” to regulate environmental humidity, HPGs facilitated directional migration of photogenerated electrons and holes to Cu_2_O's (100) and (111) planes via synergistic effects between Cu_2_O and BaTiO_3_, boosting water‐splitting efficiency. This design achieved a stable photocurrent of 224.3 µA·cm^−2^ under 10 mW cm^−2^ irradiation (**Figure** **17**A). Beyond single‐team efforts, various other catalysts,^[^ ^45^, ^163^, ^164^ ^]^ such as platinum‐titanium dioxide/polytetrafluoroethylene (Pt‐TiO_2_/PTFE),^[^ ^165^ ^]^ have also been incorporated into hydrogel systems, respectively, all demonstrating efficient hydrogen production capabilities through the synergistic effect of moisture capture and catalytic activity.

To address the remaining challenges of stability and adaptability to low‐humidity environments, Li et al.^[^ ^161^ ^]^ enhanced low‐humidity applicability by developing a hydrogel that synergizes porous media and sulfuric acid for moisture capture and storage. This system maintained stable performance at 4% RH, delivering 400 mA·cm^−2^ at 2.68 V with a hydrogen evolution rate of 745 L·day^−1^·m^−2^ under sunlight. It also achieved 574 mA·cm^−2^ and sustained high‐purity hydrogen production for over 12 days (Figure 17B). Meanwhile, Bigall et al.^[^ ^166^ ^]^ prepared cadmium selenide/cadmium sulfide (CdSe/CdS) nanorod hydrogels via ligand oxidation, achieving a hydrogen production rate tens of times higher than ligand‐stabilized nanocrystal solutions (Figure 17C)—attributed to improved charge carrier mobility in the hydrogel matrix. Notably, Yang et al.^[^ ^167^ ^]^ addressed long‐term durability by proposing a moisture‐enabled electricity generator (MEG) that couples photocatalytic hydrogen evolution with the hydrovoltaic effect to reconstruct ion concentration gradients. Comprising a hydrogel moisture‐electric generation layer, a photocatalytic layer and inert electrodes, the MEG uses photocatalytic hydrogen evolution to consume pre‐accumulated ions, restoring gradients and enabling continuous current output for over 600 h—far exceeding the lifespan of conventional systems (Figure 17D).

HPGs liberate hydrogen production from reliance on liquid water by harnessing atmospheric humidity, significantly enhancing the technology's flexibility, adaptability, and sustainability of hydrogen production technology.^[^ ^168^ ^]^ Despite these groundbreaking advances, significant challenges must be overcome before practical deployment. A major limitation is the sharp decline in moisture absorption kinetics and equilibrium water uptake under arid or low‐temperature conditions, leading to a catastrophic drop in hydrogen production rate. Furthermore, the inherent charge carrier recombination in photocatalysts results in low solar‐to‐hydrogen conversion efficiency, severely limiting the energy sustainability of the process. Additional hurdles include the long‐term stability of the gel‐catalyst interface, potential salt leakage or crystallization over extended operation, and the scalability of the material synthesis and device integration.^[^ ^160^, ^161^, ^168^ ^]^

### 5.5 Application in Advanced Greenhouses

As global climate change intensifies and water scarcity becomes increasingly severe, agricultural production faces unprecedented challenges.^[^ ^169^ ^]^ The core issues demanding urgent breakthroughs in agriculture are how to enhance water resource utilization efficiency, improve soil quality, and ensure sustainable crop growth. Atmospheric water—an unconventional water source unconstrained by geographical or hydrological conditions—offers a sustainable supply. Unlike traditional water sources, it requires no centralized water delivery systems or power infrastructure, making it a promising solution for addressing these critical challenges.^[^ ^170^ ^]^ This is particularly true for arid, sandy soils: their poor water‐holding capacity often limits moisture availability during plants’ critical growth stages, so achieving efficient regulation of soil water resources is paramount for sustainable agricultural development.

**Figure 18:**
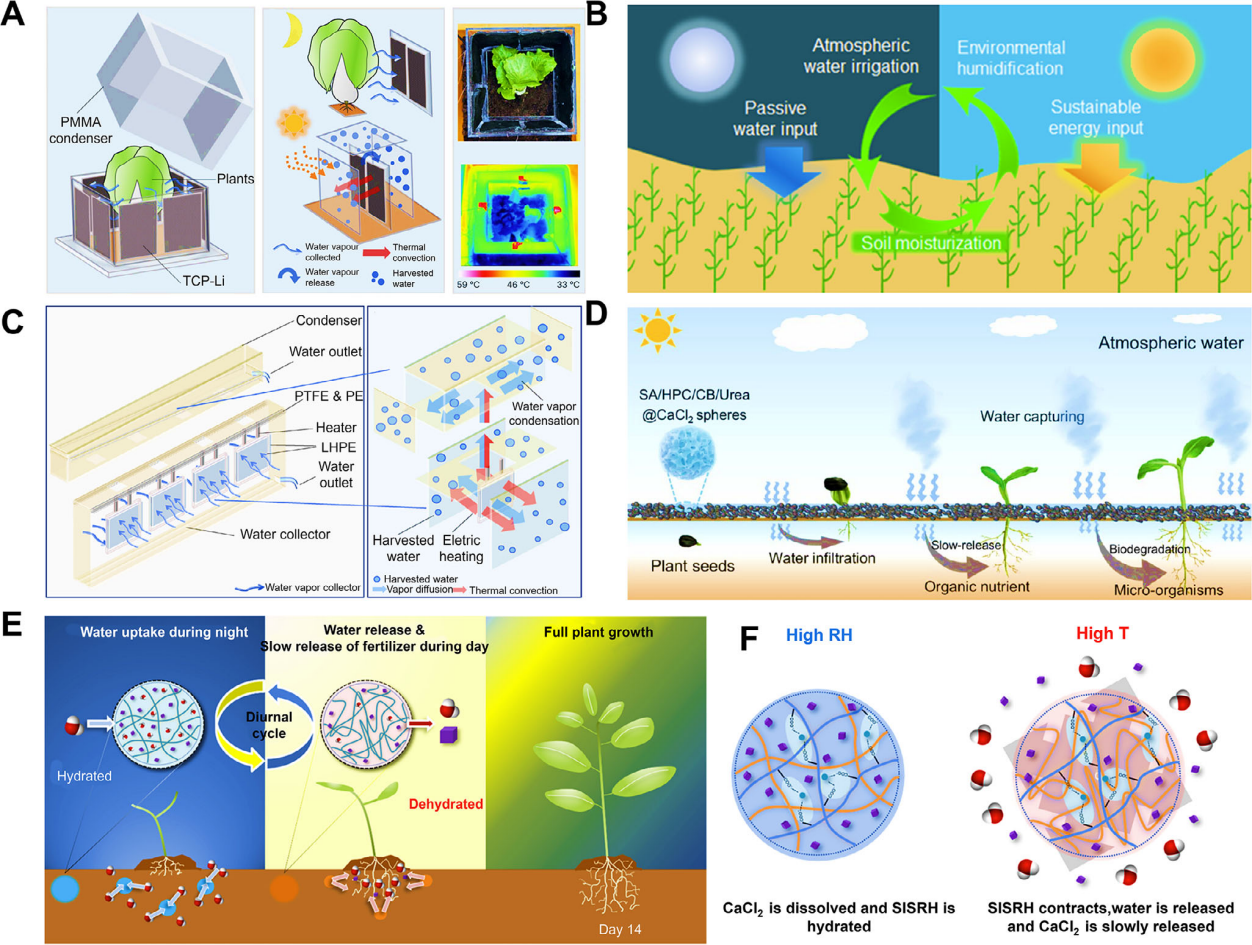
Application in Advanced Greenhouses. A) Structural schematic of the TEAD system integrated with a PMMA condenser. Reproduced with permission.^[^ ^172^ ^]^ Copyright 2024, Springer Nature. B) Schematic of atmospheric water irrigation utilizing SMAG for sustainable agriculture. Reproduced with permission.^[^ ^173^ ^]^ Copyright 2020, American Chemical Society Publications. C) Configuration and heat‐mass transfer analysis of the SWR during adsorption and desorption processes. Reproduced with permission.^[^ ^174^ ^]^ Copyright 2025, Wiley. D) Structure of SA/HPC/CB/Urea@CaCl_2_ spheres for atmospheric water harvesting and plant growth promotion. Reproduced with permission.^[^ ^176^ ^]^ Copyright 2025, American Chemical Society Publications. E) Agricultural application mechanism of PNIPAM/CaCl_2_ hydrogel: hydration during night and dehydration with water/fertilizer release during daytime. F) Slow‐release mechanism of PNIPAM/CaCl_2_ hydrogel. Reproduced with permission.^[^ ^37^ ^]^ Copyright 2024, American Chemical Society Publications.

HPGs exhibit great application potential in agriculture due to their unique water regulation mechanism. They adsorb water through hydrogen bonding and electrostatic interactions, and release it slowly via osmotic pressure gradients when the environment dries out—forming a “soil water buffer reservoir” that helps maintain stable soil moisture levels.^[^ ^171^ ^]^ Additionally, their interaction with soil particles can improve soil structure, indirectly enhancing nutrient utilization efficiency. To address the poor water retention of arid sandy soils and insufficient moisture supply during critical crop growth stages, Wang et al.^[^ ^172^ ^]^ developed a multi‐component interconnected hygroscopic porous gel (TCP‐Li), which combines excellent water absorption capacity with rapid solar‐driven water release properties. As a proof of concept, they integrated TCP‐Li into a transpiration‐evaporation adsorption device (TEAD), utilizing the gel's strong water absorption capacity to efficiently capture water vapor generated from plant transpiration and soil evaporation. TEAD operates passively: it absorbs water during high‐humidity nights and releases it for irrigation using natural sunlight during the day. Greenhouse experiments confirmed that while ensuring normal plant growth, TEAD provided an additional 87.1 g of water per plant and 1890.6 g per square meter, achieving an average water‐saving efficiency of 44.9% in irrigation (**Figure** **18**A).

Furthermore, for soil moisture regulation, some hygroscopic hydrogels possess environmental responsiveness to factors such as temperature, enabling dynamic regulation of water release behaviors based on soil conditions. Yu et al.^[^ ^173^ ^]^ reported a super moisture‐absorbent gel‐modified soil (SMAG‐soil) that enables passive atmospheric water irrigation and solar‐driven in‐chamber environmental humidification in sustainable agriculture. The atmospheric water irrigation system based on this technology first harvests atmospheric moisture for uniform irrigation. It then releases water via solar radiation to stabilize humidity in planting chambers, thereby preventing plant dehydration. Specifically, at cool, humid nights, the system absorbs and stores atmospheric water in SMAG‐soil; on warm, dry days, sunlight drives water release to compensate for moisture loss and regulate plant transpiration. Despite significant fluctuations in external RH, the system stabilizes internal humidity at ≈70%. Even under extremely low external RH, it maintains this humidity level for 20 days—demonstrating SMAG‐soil's effective humidity management capability (Figure 18B).

Beyond the direct soil moisture regulation by HPGs, the integration of hygroscopic hydrogels with agricultural facilities (such as greenhouses) further expands their application value for systematic water conservation. For example, Wang et al.^[^ ^174^ ^]^ integrated an HPG into greenhouse side window structures, further expanding its application scenarios. Composed of hydroxypropyl methylcellulose and sodium polyacrylate, this gel exhibits a water absorption capacity of 4.06 g·g^−1^ and enables rapid water release at 70  °C under solar heating. In practical applications, each square meter of the side window (with the integrated gel) can recover 5015.6 g of water, achieving a 78.78% water‐saving efficiency. It also optimizes greenhouse humidity (maintaining it at 60–80%) and temperature, reduces nighttime heat loss, and ultimately increases lettuce yield by 120% by maintaining a stable greenhouse microclimate (Figure 18C).

The core value of hygroscopic hydrogels is not limited to water regulation; their 3D network structure also provides a new approach for nutrient management. By mixing fertilizers with hydrogels or loading fertilizers within them, fertilizers gradually dissolve and release into the soil along with the moisture absorption and release process of hydrogels, thereby extending the duration of fertilizer efficiency and improving fertilizer utilization.^[^ ^175^ ^]^ For example, Ma et al.^[^ ^176^ ^]^ developed biodegradable hygroscopic hydrogel spheres from natural polysaccharides. These spheres exhibit a high water absorption capacity of 0.64–3.38 g·g^−1^, owing to the synergistic effects of their porous structure, hygroscopic salts, and hydrophilic polymer networks. Incorporating photothermal components and leveraging the low‐temperature phase‐change properties of HPC, the material enables low‐temperature desorption and rapid solar‐driven water release at a rate of 4.07 kg·m^−2^·h^−1^. In addition to stabilizing soil moisture, the hydrogel spheres facilitate controlled fertilizer release—supplying 224.6 ± 5.2 mg·kg^−1^ of nitrogen over 30 days—thereby enhancing resource‐use efficiency in agricultural applications (Figure 18D). In addition, the PNIPAM hydrogel,^[^ ^37^ ^]^ by combining with CaCl_2_, can regulate the release of nutrients required by plants while completing the diurnal rhythmic moisture absorption and release cycle. During the water release process, it only releases 59.45% of CaCl_2_ within 16 days, which not only avoids nutrient loss and soil salinization but also realizes the synergy between water supply and crop nutritional needs (Figure 18E,F).

Although these HPGs have achieved solar‐driven or temperature‐responsive water release, most hydrogels still rely on passive water release driven by soil moisture gradients, which cannot accurately meet the needs like the intelligent response triggered by crop root exudates.^[^ ^171^, ^173^ ^]^ This easily leads to mismatches between supply and demand in practical applications: for instance, during the rapid growth period of crops, water supply may be insufficient due to the slow water release of hydrogels; while during the dormant period, continuous water release may cause water waste.

### 5.6 Application in Dehumidification

**Figure 19:**
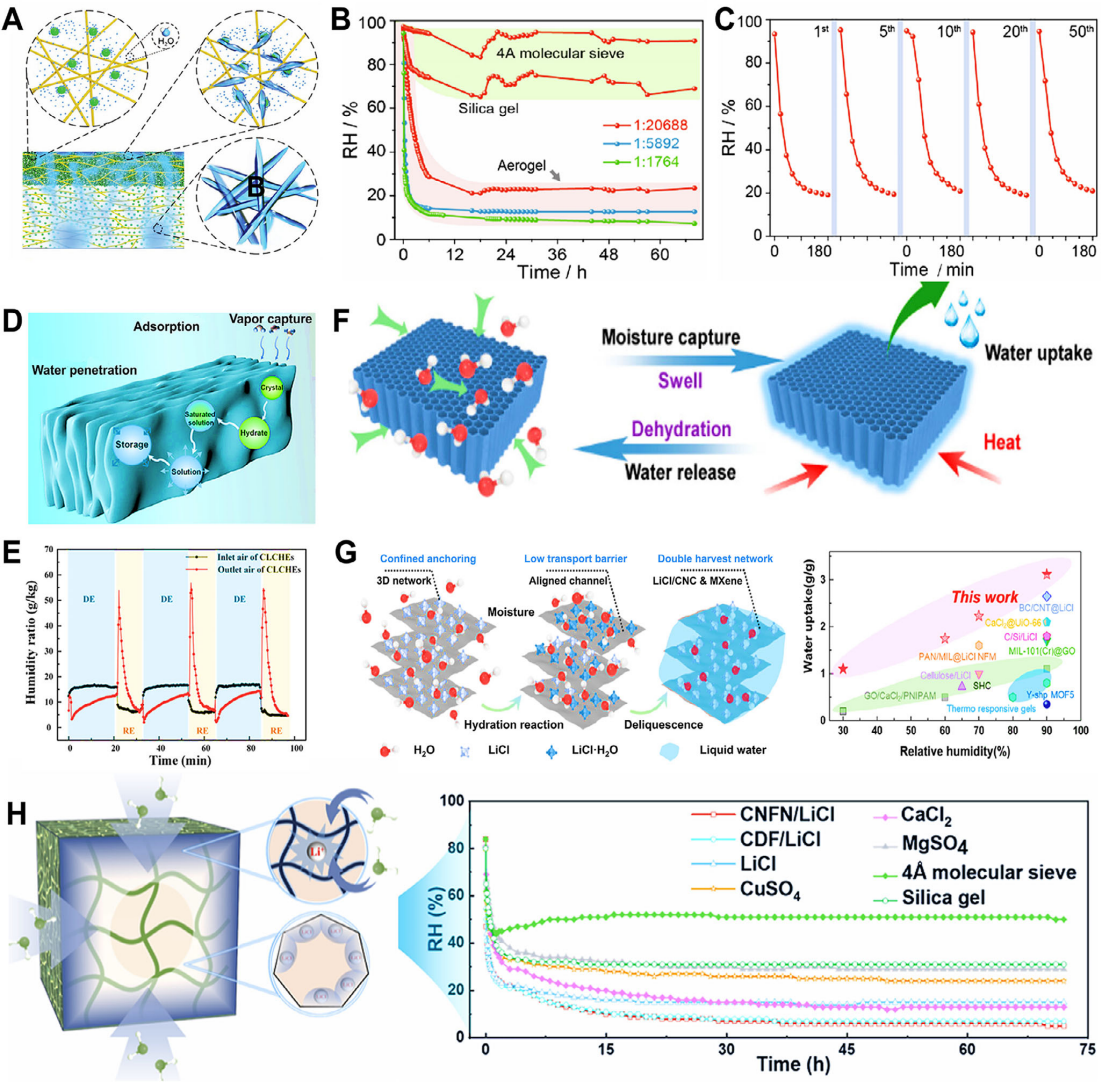
Application in Dehumidification. A) Mechanism of water vapor adsorption, liquefaction, transport, and storage in N‐MAG. B) Dehumidification performance in airtight desiccators of varying volumes using ≈1.57 cm^3^ of N‐MAG at different volume ratios. C) Recyclability of N‐MAG for repeated dehumidification at room temperature. Reproduced with permission.^[^ ^75^ ^]^ Copyright 2022, Wiley. D) Dehumidification mechanism mediated by LiCl@curdlan. Reproduced with permission. E) Cyclic dehumidification and regeneration performance under typical air conditions, evaluated by humidity ratio. Reproduced with permission.^[^ ^180^ ^]^ Copyright 2023, Cell Press. F) Moisture capture and water release mechanism of AWH. G) Sorption–desorption behavior of AWH. Reproduced with permission.^[^ ^181^ ^]^ Copyright 2024, American Chemical Society Publications. H) Comparison of water uptake between AWH and other atmospheric water harvesters. Reproduced with permission.^[^ ^34^ ^]^ Copyright 2025, American Chemical Society Publications.

Sustainable management of air humidity is crucial for humans to create a comfortable living environment. Traditional dehumidification materials struggle to meet the demand for efficient dehumidification due to issues like low adsorption efficiency and poor cyclic stability.^[^ ^177^ ^]^ By addressing these limitations, HPGs—as a new type of porous material—can spontaneously regulate environmental humidity by actively absorbing atmospheric water vapor, thanks to their large specific surface area and 3D porous structure.^[^ ^178^ ^]^ In recent years, researchers have conducted a series of studies on the structural design and performance enhancement of HPGs. Tan et al.^[^ ^179^ ^]^ reported a super‐hygroscopic multifunctional zinc oxide hydrogel. This hydrogel can absorb water vapor equivalent to ≈230% of its own weight, and upon humidity stimulation, it further exhibits synergistic changes in optical, nanocellulose network and hygroscopic lithium chloride. The interconnected nanocellulose network can quickly channel captured water into N‐MAG's internal space, preventing water accumulation near the surface and thus enabling high‐rate moisture absorption. Thanks to this structure, N‐MAG can reduce the RH from 96.7% to 28.7% within 6 h—even in a space volume over 2 × 10⁴ times its own space. Chen et al.^[^ ^75^ ^]^ developed a nanostructured moisture‐absorbing gel (N‐MAG) designed specifically for passive dehumidification. Its core (of N‐MAG) is a functional composite system consisting of a hydrophilic nats excellent performance at 30% RH, with an adsorption capacity as high as 2.65 g·g^−1^ and an adsorption rate coefficient k reaching 1.84 × 10^−4^ s^−1^, along with outstanding cyclic stability (**Figure** **19**A–C). Wang et al.^[^ ^180^ ^]^ prepared the LiCl@curdlan composite material by combining curdlan matrix with hygroscopic salt LiCl. This material exhibilectrical, and electrochemical properties. This innovation marks the first time that atmospheric moisture has been converted into a trigger for usable functional property changes (e.g., optical, electrical signals), and it can effectively reduce indoor humidity without consuming electricity (Figure 19D,E). Qu et al.^[^ ^33^ ^]^ developed a Janus‐type recyclable dehumidifying hydrogel. The synergy between its moisture absorption and dehydration functions enables recyclable dehumidification: it achieves a moisture absorption capacity of 459.3% of its own weight and a dehydration rate of 82.5%. Under dynamic conditions, it can effectively reduce the RH of the air flow from 97.1% to 39.2%.

The humidity‐regulating ability of HPGs is closely related to their internal network structure. Researchers have further improved their performance through precise structural regulation. Fu et al.^[^ ^181^ ^]^ prepared a nanocellulose‐MXene aerogel with an oriented pore structure via directional freeze‐drying technology. This aerogel further incorporates a dual‐network design, which not only enhances its water storage capacity and cyclic stability but also enables efficient atmospheric water collection. It can effectively reduce the RH in the container from 80% to 20% within 6 h (Figure 19F,G). Zhang et al.^[^ ^38^ ^]^ prepared a superhygroscopic cellulose nanofiber network/LiCl (CNFN/LiCl) aerogel with a hierarchical string‐bag structure for effective humidity control. The hierarchical structure enables efficient internal water transport, while the high hydrophilicity of cellulose promotes rapid moisture absorption from the environment—together, these features allow the aerogel to rapidly and effectively regulate environmental humidity (Figure 19H). Wang et al.^[^ ^32^ ^]^ developed a porous hygroscopic material (CASN‐Li) through 3D printing technology. This material has both macroscopically ordered mass transfer channels and micron‐scale pore structures, which synergistically accelerate internal water transport: the moisture absorption rate reaches 1.5 g·g^−1^·h^−1^ at 90% RH, and the rate at 60% RH is 2.1 times that of the solid bulk material; a sample with a size of 20 × 20 × 3 mm^3^ can reduce the humidity in a space 6750 times its own volume from 90% to 60% within 25 min.

Although HPGs have shown continuous improvements in moisture absorption capacity, rate, and cyclic stability—achieved via the optimized combination of building units and structural regulation—they still have limited adaptability to practical application scenarios. A key limitation is that existing studies are mostly based on static laboratory tests or small‐space experiments, whereas actual scenarios (such as large warehouses and industrial workshops) involve large temperature and humidity fluctuations, complex air flow, and large space scales—factors that are rarely simulated in small‐scale tests.^[^ ^177^ ^]^ This makes it difficult for HPGs to meet practical requirements in terms of efficiency, uniformity, and durability. In addition, issues such as the loss of hygroscopic components, insufficient structural cyclic stability, and high energy consumption for thermal desorption also restrict their large‐scale application.

### 5.7 Application in HPGs‐Based Electrolytes

Electrochemical energy storage technology is a cornerstone of the new energy revolution and the proliferation of intelligent electronics; however, its advancement is critically dependent on innovations in electrolyte materials.^[^ ^182^ ^]^ Current mainstream systems—including liquid and conventional solid electrolytes—face an intractable trade‐off among ionic conductivity, environmental adaptability, and safety. For instance, liquid electrolytes pose risks of leakage, volatility, and thermal runaway, while many solid electrolytes exhibit high brittleness, poor electrode contact, and drastically reduced conductivity under low ambient humidity. These limitations severely restrict their application in flexible electronics and extreme‐environment operations.^[^ ^183^ ^]^ Against this backdrop, HPGs electrolytes have emerged as a pivotal solution to the aforementioned challenges, leveraging their unique moisture absorption‐retention capabilities and integrated functional design.^[^ ^184^ ^]^ These HPGs‐based electrolytes employ a 3D porous network to capture and lock environmental moisture, which forms continuous ion migration channels. Simultaneously, hygroscopic metal salts within the gel dissociate into abundant free ions in the captured moisture. This dual synergism—between the ion migration channels (from the 3D network) and the free ions (from salt dissociation)—markedly boosts ion concentration and conductivity.^[^ ^185^ ^]^ Critically, the 3D network effectively binds ions and moisture, fundamentally resolving liquid electrolyte leakage.

**Figure 20:**
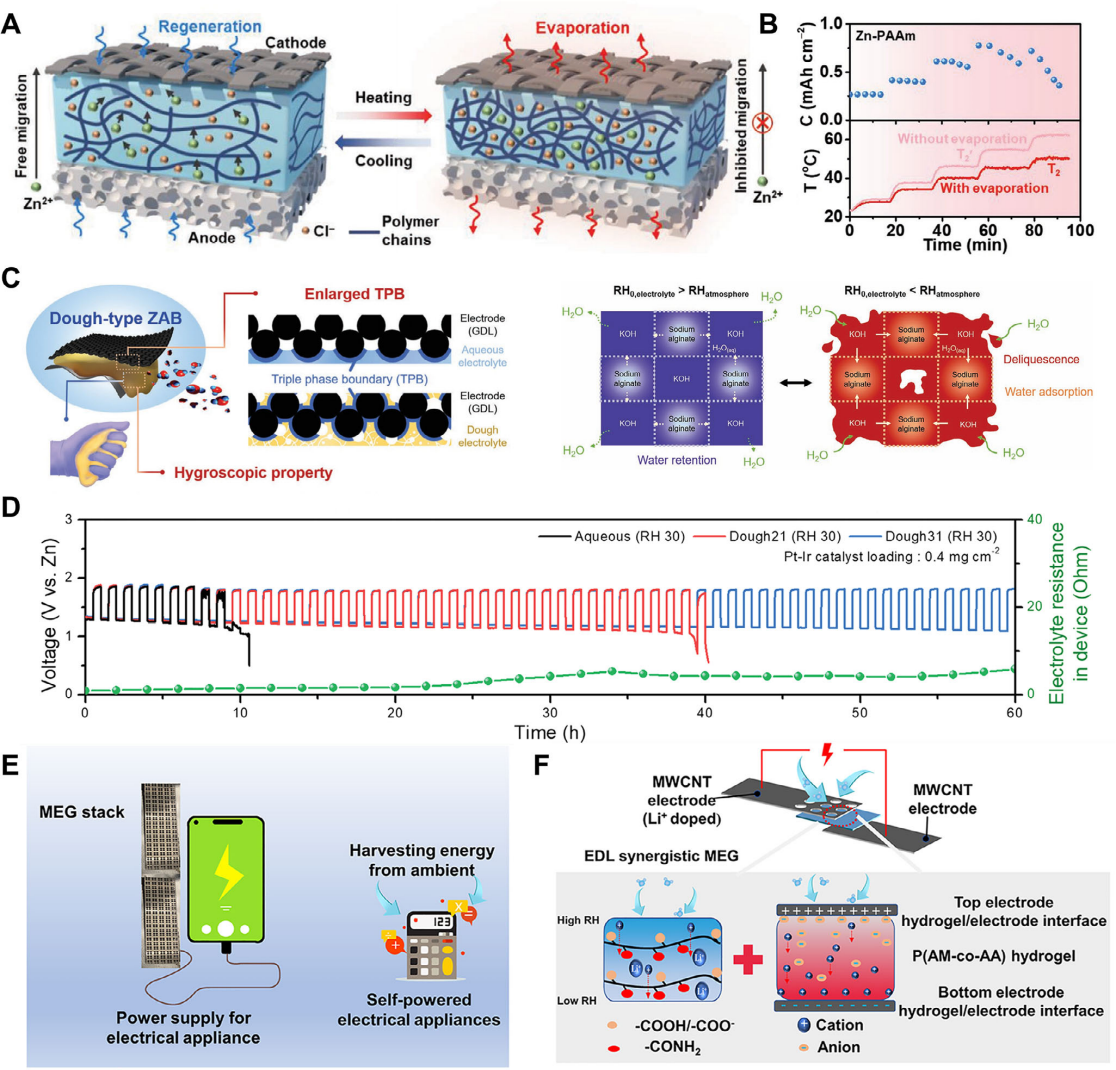
Application in HPGs‐based electrolyte. A) Working mechanism of a thermal self‐protective zinc‐ion battery employing a HPG electrolyte. B) Capacity variation of a battery using Zn‐PAAm hydrogel electrolyte and temperature evolution of MnO_2_ cathodes under transient heating, with (T_2_) and without (T_2_′) evaporative cooling. Reproduced with permission.^[^ ^186^ ^]^ Copyright 2020, Wiley. C) Configuration of the dough‐type zinc‐air battery (ZAB). D) Electrolyte resistance comparison at 30% RH and with “Dough 31″ electrolyte under 0.4 mg·cm^−2^ catalyst loading. Reproduced with permission.^[^ ^187^ ^]^ Copyright 2023, Wiley. E,F) Potential use of MEGs in power supplies and self‐powered electronic devices. Reproduced with permission.^[^ ^60^ ^]^ Copyright 2024, Cell Press.

Recent years have witnessed the successful application of HPGs‐based electrolytes in diverse systems, including supercapacitors, Zn‐ion batteries, and Zn‐air batteries, demonstrating their distinct advantages. For instance, in supercapacitors, the hygroscopic hydrogel polymer electrolyte—composed of sodium alginate (SA)/PVA/poly(acrylic acid) (PAA)‐KOH —achieves a high ionic conductivity of 0.21 S·cm^−1^ through the synergy of a multi‐component polymer network and high‐concentration KOH. It also possesses intelligent water self‐regulation capability under 20%–60% RH, significantly enhancing the environmental adaptability of supercapacitors.^[^ ^39^ ^]^ In zinc‐ion batteries, the Zn‐PAAm hydrogel^[^ ^186^ ^]^ relies on the vapor pressure regulation capability of ZnCl_2_ to achieve reversible water circulation. At high temperatures, it spontaneously “locks” the battery by evaporating internal moisture to avoid thermal runaway; at low temperatures, it absorbs moisture to regenerate and restore battery performance, offering a strategy to address the thermal runaway issue (**Figure** **20**A,B).

In zinc‐air batteries, the SA‐KOH dough‐like electrolyte,^[^ ^187^ ^]^ with its excellent interface compatibility and water retention, maintained efficient and stable operation under 30% RH and low temperature (−20  °C) environments, with a maximum power density of 160 mW/cm^2^ (Figure 20C,D). The MEG hydrogel^[^ ^60^ ^]^ achieved an open‐circuit voltage of 1.3 V under 75% RH and room temperature, an instantaneous short‐circuit current of 55.7 µA·cm^−2^, and an available power output of 2.34 µW·cm^−2^. In terms of environmental adaptability, it performs exceptionally well. When operated at 0.8 V, it remains stable even under conditions with RH below 30%. Moreover, within the temperature range of −10 to 60 °C, its output voltage can be maintained above 0.65 V (Figure 20E,F). Through molecular design and structural innovation, the HPGs‐based electrolytes have achieved the synergistic optimization of high ionic conductivity, wide environmental adaptability, and long cycle life.

These cases demonstrate that through molecular design and structural innovation, HPGs‐based electrolytes have successfully achieved the synergistic optimization of high ionic conductivity, wide environmental adaptability, and long cycle life. Collectively, these examples underscore the ability of HPGs‐based electrolytes to enable durable, high‐performance energy storage across a wide range of operating conditions. Despite these promising advances, critical challenges persist. A primary concern is performance decay under ultra‐low humidity (e.g., <15% RH), where electrolyte water loss leads to a precipitous drop in ionic conductivity and subsequent device failure. Long‐term cycling stability also requires significant improvement—especially at elevated temperatures, which accelerate electrolyte dehydration and may induce salt crystallization.^[^ ^188^, ^189^ ^]^ Future research must focus on developing novel polymer networks with enhanced water‐locking capabilities and hygroscopic salts with lower dehydration equilibrium pressures. Additionally, exploring self‐healing mechanisms and more robust electrode‐electrolyte interfaces will be crucial for achieving the required durability.

## 6 Conclusion and Perspectives

HPGs represent a highly promising platform for sustainable humidity management, leveraging their distinctive 3D porous networks, tunable swelling behaviors, and programmable moisture sorption capabilities. Recent breakthroughs in material design have enabled precise modulation of water vapor capture and release processes. This advancement has facilitated their widespread application across diverse fields, such as atmospheric water harvesting, electricity production, thermal management, fuel production, advanced greenhouses, dehumidification, and HPGs‐based electrolytes. Despite these significant advancements, several critical challenges must be overcome to further promote their practical implementation.

The primary challenge lies in the limited performance of HPGs under extreme or precision‐controlled environments—an issue inherently linked to the fundamental trade‐off between moisture management kinetics and long‐term cyclic stability. On the one hand, under arid conditions or in scenarios requiring strict humidity control (e.g., moisture‐sensitive electronics protection, precision manufacturing), HPGs often suffer from insufficient moisture uptake, slow adsorption kinetics, and delayed response to humidity fluctuations—shortcomings that directly fail to meet the high‐precision, rapid‐regulation demands of these applications. This gap underscores the urgent need to optimize the intrinsic absorption/desorption kinetics of HPGs through advanced molecular engineering strategies, including the precise incorporation of highly hydrophilic functional groups and the tailoring of network topology, to reduce the diffusion energy barrier of water vapor and enhance performance under low‐humidity conditions, On the other hand, the inherent compromise between kinetics and stability persists in constraining practical application. Pursuing high kinetic performance typically necessitates designs with open pore structures, abundant hydrophilic sites, and low diffusion barriers—yet this often comes at the expense of mechanical strength and chemical stability, leading to structural aging, loss of functional components, or network collapse over repeated adsorption–desorption cycles. Conversely, overly cross‐linked or high‐density networks enhance stability but suppress water molecule diffusion, reducing response speed and regeneration efficiency, as demonstrated in **Table** **2**. For exemple, the porous structure induced by phase separation increases the specific surface area of PBzMA gel from 50 m^2^ g^−1^ to 200 m^2^ g^−1^ and triples the moisture absorption rate at 30% RH. However, after 5 moisture absorption‐desorption cycles, the mechanical strength (compressive modulus) of the gel decreases from 2.1 to 0.8 MPa, the collapse rate of the porous structure reaches 35%, and the moisture absorption capacity at 15% RH decreases by 22%. Although the porous structure optimizes the kinetics, it impairs the network stability.^[^ ^116^ ^]^ To address this dilemma, multi‐scale collaborative design is required: at the microscale, hierarchical porous structures are constructed via phase separation and directional freezing to optimize mass transfer paths and active site distribution; at the molecular scale, gradient crosslinking and hydrophilic grafting balance chain segment mobility and network rigidity, developing dynamically adaptive networks with reversible physical crosslinking or stimuli‐responsive polymers; and at the component scale, designing heterogeneous composite structures with precise integration of high‐kinetic components and high‐stability frameworks. Meanwhile, leveraging machine learning and high‐throughput computation‐aided design is expected to more accurately balance the “mass transfer‐stability” competitive relationship within the materials.

**Table 2:** Performance Comparison Table of HPGs under Low‐Humidity.

Material	Water absorption capability(RH)	Desorption capacity (T/RH)	Cyclic stability (RH)
SA/HPMC/Li^+^/CNT^[^ ^56^ ^]^	0.70‐0.94 g/g (20–30%)	90% (30 °C)	25 (30%), >95%
TiN/HPMC/LiCl^[^ ^28^ ^]^	1.18‐1.54 g/g (15–30%)	95% (20‐30%)	15 (60%), >97%
KGM/HPC/LiCl^[^ ^190^ ^]^	0.64‐0.96 g/g (15–30%)	0.56‐0.82 g/g (15‐30%)	14‐24 (15‐30%)
PAM/LiCl^[^ ^62^ ^]^	0.26‐1.1‐1.5 g/g (10–20–30%)	0.51 g/g (20%)	15 (20%)
ZHPC/LiCl^[^ ^90^ ^]^	0.86‐1.32 g/g (15–30%)	90% (60 °C)	20, 100%
PDMAPS/LiCl^[^ ^70^ ^]^	0.62 g/g (30%)	0.5 g/g (30%)	10 (30%), 100%
P(NIPAM‐co‐DMAPS)/LiCl^[^ ^91^ ^]^	0.23‐0.48 g/g (15–30%)	0.36 g/g (30%)	12 (30%)
HPC/LiCl^[^ ^93^ ^]^	0.5‐0.8 g/g (15–30%)	0.0079‐0.0191 g/g/day (15‐30%)	24‐36 (15‐30%)
LiCl/rGO‐SA^[^ ^106^ ^]^	1.01‐1.52 g/g (15–30%)	0.8 g/g (70 °C/14%)	10 (30%), 95%
PNIPAM/PNMA/CNTs^[^ ^30^ ^]^	0.43‐0.89 g/g (15–30%)	40 °C	–
CNF/SNF/LiCl^[^ ^191^ ^]^	0.90‐1.43 g/g (15–25%)	60 °C	36 (30%)
HPC/SA/DAL/LiCl^[^ ^94^ ^]^	0.62‐0.872‐1.74 g/g/h (15–20–30%)	0.00198 g/g/h (65 °C/30%)	36 /day

Beyond fundamental material‐level constraints (kinetics‐stability trade‐off), HPGs also face device‐ and application‐level challenges: monofunctionality and insufficient system integration. Most existing studies focus on improving individual performance parameters such as moisture absorption capacity or desorption rate, while overlooking the need for multifunctional synergy and dynamic regulation in practical applications. For instance, in intelligent humidity‐control systems, HPGs are expected to integrate moisture capture/release, humidity sensing, and self‐regulation—but current systems rely on external sensors and controllers, increasing complexity. To achieve truly optimized performance, it is essential to overcome the cooperative design bottleneck between materials and devices. On one hand, material‐level development should aim to create gel systems with rapid response, stable cycling, and adaptive environmental characteristics. On the other hand, device‐level design requires holistic considerations of mass transfer structures, interfacial compatibility, and mechanical adaptability. For example, flexible modules featuring hierarchical flow channels and buffered interfaces should be designed to accommodate the dynamic swelling/shrinking of HPGs, thereby avoiding structural failure caused by stress concentration and ensuring long‐term device reliability.

Furthermore, safety concerns regarding the leakage and toxicity of certain hygroscopic salts (e.g., LiCl) restrict their use in indoor and wearable applications. Future efforts should prioritize bio‐based polymer matrices (e.g., cellulose, chitosan) and low‐toxicity hygroscopic agents (e.g., green ionic liquids) to enhance biosafety and environmental compatibility. Moreover, most current studies are confined to lab‐scale tests under static conditions, failing to replicate the heterogeneous, dynamic demands of real‐world environments (e.g., industrial plants, greenhouses). Bridging this lab‐to‐application gap requires developing HPGs‐based environmentally adaptive gel systems with dynamically tunable sorption properties, integrated with macrostructural innovations (e.g., hierarchically porous architectures, flexible device engineering) to enable efficient humidity management across large, complex spaces.

HPGs demonstrate considerable transformative potential in enabling sustainable technologies for energy conservation, water security, and ecological protection. Advancing these materials from laboratory prototypes to industrial‐scale applications necessitates overcoming persistent challenges in stability, environmental adaptability, and multifunctional integration. Achieving this goal will require interdisciplinary collaboration among materials science, chemical engineering, and environmental science—with a focus on fostering innovations in molecular design, advanced fabrication techniques, and application‐specific system engineering. With sustained research and development, HPGs are anticipated to become core elements of next‐generation sustainable humidity management systems, thereby playing a critical role in facilitating the transition toward a green and low‐carbon society.

## Conflict of Interest

The authors declare no conflict of interest.
